# Trends in Self-Reported Adherence to Healthy Lifestyle Behaviors Among US Adults, 1999 to March 2020

**DOI:** 10.1001/jamanetworkopen.2023.23584

**Published:** 2023-07-14

**Authors:** Yue Li, Peng-Fei Xia, Ting-Ting Geng, Zhou-Zheng Tu, Yan-Bo Zhang, Han-Cheng Yu, Ji-Juan Zhang, Kunquan Guo, Kun Yang, Gang Liu, Zhilei Shan, An Pan

**Affiliations:** 1 Department of Epidemiology and Biostatistics, Ministry of Education Key Laboratory of Environment and Health, School of Public Health, Tongji Medical College, Huazhong University of Science and Technology, Wuhan, China; 2 Department of Nutrition and Food Hygiene, School of Public Health, Institute of Nutrition, Fudan University, Shanghai, China; 3 Department of Endocrinology, Affiliated Dongfeng Hospital, Hubei University of Medicine, Shiyan, China; 4 Department of Nutrition and Food Hygiene, Hubei Key Laboratory of Food Nutrition and Safety, School of Public Health, Tongji Medical College, Huazhong University of Science and Technology, Wuhan, China

## Abstract

**Importance:**

Adherence to a healthy lifestyle is associated with lower risks of adverse outcomes. However, trends in multiple lifestyle factors and overall healthy lifestyle status among US adults in recent years are unknown.

**Objective:**

To examine trends in multiple lifestyle factors and overall healthy lifestyle among US adults.

**Design, Setting, and Participants:**

This serial cross-sectional study used nationally representative data from 10 National Health and Nutrition Examination Survey (NHANES) cycles (nine 2-year cycles from 1999 to 2016 and 1 combined cycle from 2017 to March 2020) among adults 20 years or older. Data were analyzed from December 10, 2021, to January 11, 2023.

**Exposure:**

Survey cycle.

**Main Outcomes and Measures:**

Five healthy lifestyle factors: never smoking, moderate or lighter alcohol consumption (for women: ≤7 drinks/wk; for men: ≤14 drinks/wk), healthy diet (Healthy Eating Index–2015 scores ≥60.0), sufficient physical activity (≥150 min/wk of equivalent moderate physical activity), and healthy weight (body mass index [calculated as weight in kilograms divided by height in meters squared] 18.5-24.9).

**Results:**

A total of 47 852 adults were included in this study. The weighted mean [SE] age was 47.3 [0.2] years; 24 539 (weighted proportion, 51.5%) were women. From the 1999-2000 cycle to the 2017 to March 2020 cycle, the estimated prevalence of the 5 lifestyle factors showed divergent trends, with increasing prevalence of never smoking (from 49.4% [95% CI, 46.4%-52.4%] to 57.7% [95% CI, 55.5%-59.9%]; difference, 8.2% [95% CI, 4.5%-12.0%]), healthy diet (from 19.3% [95% CI, 16.0%-22.6%] to 24.5% [95% CI, 21.5%-27.5%]; difference, 5.2% [95% CI, 0.8%-9.7%]), and sufficient physical activity (from 55.7% [95% CI, 51.8%-59.6%] to 69.1% [95% CI, 67.2%-71.1%]; difference, 13.4% [95% CI, 9.0%-17.8%]), while prevalence of healthy weight decreased from 33.1% (95% CI, 30.5%-35.6%) to 24.6% (95% CI, 22.6%-26.7%; difference, −8.4% [95% CI, −11.8% to −5.1%]) (all *P* < .001 for trend). Meanwhile, there was no significant trend in moderate or lighter alcohol consumption. Overall, the estimated prevalence of at least 4 healthy lifestyle factors increased from 15.7% (95% CI, 12.8%-18.7%) to 20.3% (95% CI, 17.8%-22.7%; difference, 4.5% [95% CI, 0.7%-8.4%]; *P* < .001 for trend). Disparities in healthy lifestyle were widened by age group, with little improvement among adults 65 years and older (difference, 0.04% [95% CI, −4.28% to 4.35%]). There were persistent disparities in healthy lifestyle by race and ethnicity, educational level, and income level.

**Conclusions and Relevance:**

The findings of this cross-sectional study of NHANES data over a 22-year period suggest diverse change patterns across 5 healthy lifestyle factors and a modest improvement in overall lifestyle existed among US adults, with worsening or persistent disparities in lifestyle.

## Key Points

**Question:**

What were trends in lifestyle factors among US adults from the 1999-2000 cycle to the combined 2017 to March 2020 cycle of the National Health and Nutrition Examination Survey?

**Findings:**

In this cross-sectional study including 47 852 adults, improvements were observed in smoking habits, diet quality, and physical activity levels, but with a decrease in healthy weight and no significant change in moderate or less alcohol consumption. Overall, the prevalence of having at least 4 factors at a healthy level increased from 16% to 20%, but with worsening disparities by age group and persistent disparities by race and ethnicity and socioeconomic level.

**Meaning:**

These findings suggest that efforts are still warranted to improve lifestyle in US adults, with attention on equity.

## Teaser

This cross-sectional study assesses trends in multiple lifestyle and combined healthy lifestyle factors among US adults using recently released data from the National Health and Nutrition Examination Survey for cycles between 1999 and March 2020.

## Introduction

Adhering to healthy lifestyle factors, including avoiding tobacco use, limiting alcohol consumption, maintaining a healthy diet, engaging in sufficient physical activity, and maintaining healthy body weight, is a cost-effective strategy for preventing noncommunicable diseases (NCDs) suggested by the World Health Organization.^1^ Mounting evidence has shown that maintaining or improving a healthy lifestyle is associated with lower risks of various NCDs and mortality and with higher life expectancy.^2,3,4,5,6^ Therefore, a description of trends in lifestyle factors could provide important data for policy makers and relevant stakeholders to understand and forecast the health status of the population and facilitate policy making to improve population health.

A number of studies have described the population-level trends in individual lifestyle factors among US adults in recent years, including trends in smoking habits (1965-2018 and 2002-2016),^7,8^ alcohol consumption (2000-2016 and 2011-2017),^9,10^ diet quality (1999-2016),^11^ physical activity level (2007-2016 and 2008-2018),^12,13^ and body mass index (BMI) (2007-2016 and 1999-2018).^14,15^ However, few studies simultaneously focused on trends in multiple lifestyle factors, thus a complete picture of the population health status is lacking, given that lifestyle factors are interrelated with each other. Hence, it is essential to understand the current status and long-term changes in overall lifestyle at the population level. Several studies^16,17,18^ have reported trends of overall lifestyle among US adults prior to 2007, but no study has reported trends of overall lifestyle during the past decade since 2010. To address these gaps, we used the recently released data from the National Health and Nutrition Examination Survey (NHANES) to examine trends in multiple lifestyle factors as well as combined healthy lifestyle factors among US adults from the 1999-2000 cycle to the combined cycle from 2017 to March 2020.

## Methods

### Study Population

The NHANES is a series of cross-sectional surveys with a complex, multistage probability sample design conducted by the National Center for Health Statistics (NCHS) to obtain health-related information about the civilian noninstitutionalized population in the US. Details of the study design, protocol, and data collection have been described elsewhere.^19^ Between the 1999-2000 and the combined 2017 to March 2020 cycles (nine 2-year cycles from 1999 to 2016 and 1 combined cycle due to the COVID- 2019 pandemic from 2017 to March 2020),^20^ the unweighted total response rates ranged from 51% to 84% for the interviewed samples and from 47% to 80% for the examined samples.^21^ The NHANES protocol was approved by the NCHS Research Ethics Review Board, and all participants provided written consent. This study followed the Strengthening the Reporting of Observational Studies in Epidemiology (STROBE) reporting guideline.

In the present analyses, we included adults 20 years or older who had complete data for smoking, alcohol consumption, diet, physical activity, and BMI from the 1999-2000 cycle through the 2017 to March 2020 cycle. In the main analysis, we only used the first 24-hour dietary recall data to evaluate diet quality and did not use the second 24-hour dietary recall, which was available since the 2003-2004 cycle. This was mainly because distributions of dietary intake were not comparable from single vs multiple dietary recalls,^22^ and the survey method was different between the first dietary recall (in-person) and second recall (telephone).^23^

### Data Collection

Data were collected during the household interview and a study visit in the mobile examination center (MEC). Information on race and ethnicity was self-reported through standardized questionnaires according to the fixed classifications provided by the NCHS (Mexican American, non-Hispanic Black, non-Hispanic White, other Hispanic, or other [including non-Hispanic Asian and individuals of >1 race]). This information was collected to report current status and changes in prevalence of lifestyle factors by race and ethnicity. In line with a previous study conducted in the NHANES,^16^ we included 5 lifestyle factors: smoking, alcohol consumption, diet quality, physical activity, and BMI. Information on smoking was obtained by questions about whether the participant smoked at least 100 cigarettes in life, whether they smoked at the time of the survey, and numbers of cigarettes, pipes, or cigars smoked during the past 30 days. Alcohol consumption was assessed by self-reported drinking frequency and drinking quantity over the past year. Diet information was assessed using one 24-hour dietary recall conducted in person in the MEC. In the dietary recall interview, the participant reported all foods and beverages consumed during the prior 24 hours. For physical activity, the questionnaire changed from a specific Physical Activity and Physical Fitness Questionnaire before the 2007-2008 cycle to the Global Physical Activity Questionnaire thereafter. Both questionnaires accessed the duration of physical activity from different domains. Briefly, the former assessed minutes of physical activity during the past 30 days from the household, transportation, and moderate to vigorous leisure time; the latter measured minutes of physical activity in a typical week from moderate to vigorous work, transportation, and moderate to vigorous leisure time. Body weight and height were measured using a digital weight scale and a fixed stadiometer respectively, following standard procedures (eg, wearing requirement and standing posture) at the MEC. Details of the measurements of the 5 lifestyle factors can be found elsewhere.^24^

### Outcomes

The primary outcomes were 5 lifestyle factors and combined healthy lifestyle factors. To capture more detailed information in lifestyle factors, individual lifestyle factors were first classified into multiple levels. Smoking status was categorized as never smoking (having smoked <100 cigarettes in life), former smoking (having smoked ≥100 cigarettes in life but not smoking currently), and current smoking (smoked cigarettes every day or some days at the time of the survey) of 1 to 14, 15 to 24, and at least 25 cigarettes per day.^25^ Original alcohol consumption data were transferred into drinks (14 g of ethanol per drink) per week. Alcohol consumption was categorized as none, light (≤3 drinks/wk), moderate (>3 to 7 drinks/wk for women or >3 to 14 drinks/wk for men), and heavy (>7 drinks/wk for women or >14 drinks/wk for men).^26^ Diet quality was assessed by the Healthy Eating Index (HEI)–2015, which reflected adherence to the 2015 to 2020 Dietary Guidelines for Americans.^27^ The HEI-2015 score ranged from 0 (no adherence) to 100 (best adherence)^28^ and was categorized as less than 40.0, 40.0 to 49.9, 50.0 to 59.9, and 60.0 or greater.^29^ The scoring method of the HEI-2015 is described in eTable 1 in Supplement 1. Physical activity from the household and transportation was defined as moderate activity according to the NHANES guidelines.^12^ The minutes of vigorous activity were equal to the doubled minutes of moderate activity.^30^ Total amount of physical activity was calculated as the minutes of equivalent moderate physical activity per week of all domains, and 4 levels of physical activity were created: 0, 0.1 to 149.9, 150.0 to 300.0, and greater than 300.0 min/wk.^30,31^ We calculated BMI as weight in kilograms divided by height in meters squared and categorized it into 6 levels: less than 18.5, 18.5 to 24.9, 25.0 to 29.9, 30.0 to 34.9, 35.0 to 39.9, and 40.0 or greater.^32^ Individual lifestyle factors were then defined as dichotomized variables with healthy and unhealthy levels, and healthy levels were described as follows: never smoking,^33^ moderate or lighter alcohol consumption (≤7 drinks/wk for women or ≤14 drinks/wk for men),^26^ healthy diet (HEI-2015 scores of ≥60.0),^29^ sufficient physical activity (≥150 min/wk),^31^ and healthy weight (BMI, 18.5-24.9).

For each healthy lifestyle factor, the participant who met the criterion for a healthy level received a score of 1; others received 0. The healthy lifestyle score was defined as the sum of all 5 scores and ranged from 0 to 5, with higher scores indicating healthier lifestyle. Since the number of participants with the highest lifestyle score was small across the 10 cycles (ranging from 96 to 221) (eTable 2 in Supplement 1), participants with 4 or 5 healthy lifestyle factors were combined into 1 group and defined as having a healthy lifestyle.^6^

Secondary outcomes were trends in 5 healthy lifestyle factors and healthy lifestyle by major sociodemographic subgroups (age, sex, race and ethnicity, educational level, and income) assessed by standardized questionnaires. The subgroups were chosen since previous research in US adults documented the co-occurrence of healthy lifestyle factors among sociodemographic strata.^34,35,36^

### Statistical Analysis

Data were analyzed from December 10, 2021, to January 11, 2023. In all analyses, survey procedures were used to account for dietary sample weights,^37^ stratification, and clustering of the complex sampling design to ensure nationally representative estimates. Characteristics of participants by different cycles were presented as numbers (percentages) for categorical variables and compared using the Rao-Scott χ^2^ test.

To reflect the actual change in lifestyle factors, crude weighted prevalence and 95% CIs of individual lifestyle factors and combined healthy lifestyle factors were estimated by cycle.^38^ Crude trends in lifestyle factors were assessed by treating the survey cycle as a continuous variable in logistic regression models. Absolute differences in weighted prevalence were calculated between the first and the last cycle. Stratified analyses were performed by age group (20-34, 35-49, 50-64, and ≥65 years), sex (women and men), race and ethnicity (Mexican American, non-Hispanic Black, and non-Hispanic White), educational level (less than high school, high school or equivalent, and college or above),^11^ and income (ratio of family income to poverty: <1.30 [low income], 1.30-3.49 [middle income], and ≥3.50 [high income]).^11^ Trends for other races and ethnicities were not analyzed because it was hard to calculate reliable estimates for the group across all NHANES cycles.^39^ Interactions were performed by including multiplicative terms of the survey cycle with each stratified variable to assess the heterogeneity of trends over time among subgroups. *P* values for interactions were further adjusted with Benjamini–Hochberg false discovery rate (FDR) correction.^40^ To determine whether trends were due to sociodemographic shifts, additional analyses were conducted with adjustments for age group and all sociodemographic variables. In sensitivity analyses, to further incorporate the data of 2 dietary recalls since the 2003-2004 cycle, trends in diet quality were assessed in adults with 2 valid recalls between the 2003-2004 cycle and the 2017 to March 2020 cycle. Similarly, due to the change in the physical activity questionnaire since the 2007-2008 cycle, trends in physical activity were accessed separately from the 1999-2000 to 2005-2006 cycles and from the 2007-2008 cycle to the 2017 to March 2020 cycle. Participants with missing data on educational level (n = 38) and income (n = 4065) were excluded in the corresponding stratified analyses and imputed using multiple imputation in multivariable analyses. All analyses were conducted with SAS, version 9.4 (SAS Institute Inc), and 2-tailed *P* < .05 was considered statistically significant.

## Results

### Participant Characteristics

Of the 47 852 US adults included in the analyses, the weighted mean (SE) age was 47.3 (0.2) years; 24 539 (weighted proportion, 51.5%) were women and 23 313 (weighted proportion, 48.5%) were men. In terms of race and ethnicity, 8113 (weighted proportion, 8.0%) were Mexican American, 10 181 (weighted proportion, 11.0%) were non-Hispanic Black, and 21 529 (weighted proportion, 68.9%) were non-Hispanic White. Significant differences were observed in the distribution of participants by age and educational level groups over time (Table 1).

**Table 1.** Characteristics of US Adults 20 Years or Older, 1999 to March 2020

Characteristic	NHANES cycle, No. of participants (weighted %)^a^										*P* value
	1999-2000 (n = 3967)	2001-2002 (n = 4295)	2003-2004 (n = 4120)	2005-2006 (n = 4184)	2007-2008 (n = 5001)	2009-2010 (n = 5196)	2011-2012 (n = 4372)	2013-2014 (n = 4740)	2015-2016 (n = 4652)	2017 to March 2020 (n = 7325)	
Age group, y											
20-34	1056 (29.5)	1179 (27.3)	1112 (29.2)	1268 (27.5)	1166 (27.5)	1296 (27.3)	1209 (27.9)	1251 (27.9)	1178 (27.5)	1704 (27.6)	<.001
35-49	964 (31.1)	1178 (33.7)	956 (28.7)	1064 (30.1)	1252 (30.0)	1350 (28.6)	1028 (25.6)	1209 (25.6)	1124 (24.5)	1721 (24.6)	
50-64	892 (21.3)	952 (23.0)	852 (24.5)	903 (24.7)	1307 (25.8)	1306 (26.6)	1169 (28.5)	1230 (27.7)	1212 (26.7)	2119 (26.5)	
≥65	1055 (18.0)	986 (16.0)	1200 (17.6)	949 (17.7)	1276 (16.6)	1244 (17.5)	966 (18.0)	1050 (18.8)	1138 (21.4)	1781 (21.3)	
Sex											
Women	2100 (51.7)	2238 (51.4)	2131 (51.8)	2173 (51.7)	2536 (53.0)	2634 (51.0)	2154 (50.6)	2443 (51.1)	2389 (51.5)	3741 (51.4)	.87
Men	1867 (48.3)	2057 (48.6)	1989 (48.2)	2011 (48.3)	2465 (47.0)	2562 (49.0)	2218 (49.4)	2297 (48.9)	2263 (48.5)	3584 (48.6)	
Race and ethnicity											
Mexican American	1058 (7.2)	903 (6.6)	810 (7.5)	834 (7.7)	858 (8.3)	956 (8.4)	409 (7.5)	625 (9.0)	815 (8.7)	845 (8.3)	.08
Non-Hispanic Black	715 (10.3)	800 (10.4)	792 (11.0)	919 (11.1)	1026 (11.1)	908 (11.1)	1163 (11.3)	942 (11.1)	968 (10.6)	1948 (11.3)	
Non-Hispanic White	1814 (70.5)	2275 (73.7)	2242 (73.9)	2143 (73.8)	2386 (70.9)	2547 (69.6)	1716 (67.7)	2128 (66.5)	1617 (65.4)	2661 (63.0)	
Other^b^	380 (12.0)	317 (9.3)	276 (7.6)	288 (7.5)	731 (9.7)	785 (11.0)	1084 (13.5)	1045 (13.3)	1252 (15.3)	1871 (17.5)	
Educational level^c^											
Less than high school	1496 (24.3)	1237 (18.2)	1156 (16.9)	1108 (16.4)	1509 (19.6)	1432 (18.3)	973 (15.5)	946 (14.4)	1052 (13.2)	1253 (10.1)	<.001
High school or equivalent	900 (26.3)	1002 (24.8)	1029 (26.5)	1016 (25.3)	1228 (25.5)	1205 (22.6)	923 (20.1)	1060 (21.9)	1033 (21.1)	1771 (27.5)	
College or above	1561 (49.4)	2054 (57.0)	1932 (56.6)	2060 (58.3)	2260 (54.9)	2550 (59.1)	2473 (64.4)	2733 (63.7)	2566 (65.7)	4295 (62.4)	
Family income-poverty ratio level^d^											
<1.30	1028 (23.3)	1018 (19.6)	1107 (20.8)	1016 (17.1)	1359 (20.6)	1526 (21.2)	1381 (24.1)	1473 (24.4)	1333 (20.7)	1766 (19.1)	.29
1.30-3.49	1312 (35.5)	1566 (35.1)	1540 (37.2)	1577 (37.3)	1780 (33.8)	1795 (35.3)	1409 (34.0)	1532 (34.5)	1703 (36.7)	2506 (34.0)	
≥3.50	1085 (41.1)	1438 (45.3)	1258 (42.0)	1420 (45.6)	1431 (45.6)	1423 (43.4)	1256 (41.8)	1411 (41.2)	1180 (42.6)	2158 (46.9)	

Abbreviation: NHANES, National Health and Nutrition Examination Survey.

^a^ Percentages were adjusted for NHANES survey weights.

^b^ Includes non-Hispanic Asian, other Hispanic, and more than 1 race.

^c^ Data were missing for 38 participants.

^d^ Data were missing for 4065 participants. The variable was calculated by dividing family income by the poverty guidelines. A higher ratio represents a higher level of income.

### Trends in Individual Lifestyle Factors

**Figure 1.**
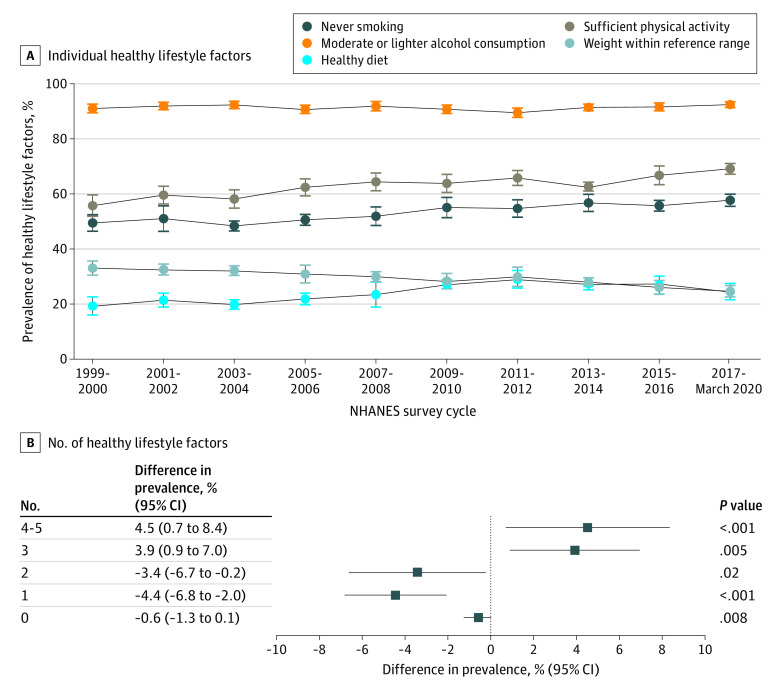
Trends in Estimated Prevalence of Healthy Lifestyle Factors Among US Adults 20 Years or Older, 1999 to March 2020 Crude trends in prevalence of healthy lifestyle factors (A) and changes in estimated prevalence of specific numbers of healthy lifestyle factors (B) are shown. Healthy lifestyle factors included never smoking, moderate or lighter alcohol consumption (≤14 drinks/wk for men or ≤7 drinks/wk for women), healthy diet (a Healthy Eating Index–2015 score ≥60.0), sufficient physical activity (≥150 min/wk moderate to vigorous intensity physical activity), and healthy weight (body mass index [calculated as weight in kilograms divided by height in meters squared] 18.5-24.9). Data were adjusted for National Health and Nutrition Examination Survey survey weights. Error bars indicate 95% CIs. In some cases, the 95% CIs may cross over 0 even if the *P* value for trend is significant because the 95% CIs are based on data from the 1999-2000 cycle and the combined cycle from 2017 to March 2020, and the *P* value is based on data across 10 cycles.

Divergent trends were observed among 5 healthy lifestyle factors (Figure 1A). From the 1999-2000 to the 2017 to March 2020 cycles, the estimated prevalence of never smoking increased from 49.4% (95% CI, 46.4%-52.4%) to 57.7% ([95% CI, 55.5%-59.9%]; difference, 8.2% [95% CI, 4.5%-12.0%]), that of healthy diet increased from 19.3% (95% CI, 16.0%-22.6%) to 24.5% (95% CI, 21.5%-27.5%; difference, 5.2% [95% CI, 0.8%-9.7%]), and that of sufficient physical activity increased from 55.7% (95% CI, 51.8%-59.6%) to 69.1% (95% CI, 67.2%-71.1%; difference, 13.4% [95% CI, 9.0%-17.8%]), while that of healthy weight decreased from 33.1% (95% CI, 30.5%-35.6%) to 24.6% (95% CI, 22.6%-26.7%; difference, −8.4% [95% CI, −11.8% to −5.1%]) (all *P* < .001 for trend). During this period, the estimated prevalence of moderate or lighter alcohol consumption remained stable from 91.0% (95% CI, 89.4%-92.6%) to 92.4% (95% CI, 91.4%-93.4%; *P* = .52 for trend). In addition, the 2011-2012 cycle had the highest prevalence of healthy diet (29.0% [95% CI, 25.8%-32.2%]). In sensitivity analyses, a similar increase in healthy diet based on 2 diet recalls was observed from the 2003-2004 cycle to the 2017 to March 2020 cycle (eTable 3 in Supplement 1), and increases in sufficient physical activity remained consistent from the 1999-2000 to 2005-2006 cycles and from the 2007-2008 to the 2017 to March 2020 cycles (eTable 4 in Supplement 1). Moreover, the adjustment of age or all sociodemographic characteristics did not alter the results (eTable 5 in Supplement 1).

Trends in the prevalence of multiclass lifestyle factors are shown in Table 2 and eTable 4 in Supplement 1. From the 1999-2000 to the 2017 to March 2020 cycles, decreases were observed for current smoking of 15 to 24 and 25 or more cigarettes/d, no alcohol consumption, and overweight (BMI 25.0-29.9), while increases were observed in light drinking, class I obesity (BMI 30.0-34.9), class II obesity (BMI 35.0-39.9), and class III obesity (BMI ≥40.0).

**Table 2.** Crude Trends in Estimated Prevalence of Lifestyle Factors Among US Adults 20 Years or Older, 1999 to March 2020

Lifestyle factor	Estimated prevalence of lifestyle factors, % (95% CI)^a^										*P* value for trend	Difference, 2017 to March 2020 vs 1999-2000 (95% CI)^b^
	1999-2000 (n = 3967)	2001-2002 (n = 4295)	2003-2004 (n = 4120)	2005-2006 (n = 4184)	2007-2008 (n = 5001)	2009-2010 (n = 5196)	2011-2012 (n = 4372)	2013-2014 (n = 4740)	2015-2016 (n = 4652)	2017 to March 2020 (n = 7325)		
Smoking												
Never	49.4 (46.4 to 52.4)	51.0 (46.4 to 55.7)	48.3 (46.6 to 50.1)	50.6 (48.6 to 52.5)	51.9 (48.5 to 55.2)	55.0 (51.4 to 58.7)	54.7 (51.5 to 57.9)	56.7 (53.6 to 59.8)	55.7 (53.8 to 57.7)	57.7 (55.5 to 59.9)	<.001	8.2 (4.5 to 12.0)
Former	26.1 (23.6 to 28.6)	25.4 (22.3 to 28.4)	26.8 (24.8 to 28.7)	24.9 (22.6 to 27.2)	24.5 (23.1 to 26.0)	25.0 (22.4 to 27.7)	24.8 (21.7 to 27.9)	24.5 (22.0 to 27.0)	26.0 (23.9 to 28.1)	25.6 (24.0 to 27.2)	.74	−0.5 (−3.5 to 2.4)
Current, cigarettes/d												
1-14	11.2 (9.7 to 12.7)	11.7 (9.7 to 13.6)	12.0 (10.4 to 13.5)	11.5 (10.5 to 12.4)	13.3 (12.3 to 14.2)	11.5 (10.4 to 12.6)	13.3 (11.9 to 14.7)	12.6 (11.4 to 13.9)	12.2 (10.5 to 13.9)	10.4 (9.1 to 11.8)	.63	−0.8 (−2.8 to 1.3)
15-24	8.3 (6.7 to 9.9)	9.0 (7.9 to 10.2)	8.5 (7.3 to 9.8)	8.9 (7.7 to 10.2)	6.9 (5.5 to 8.3)	6.6 (5.1 to 8.0)	5.1 (3.8 to 6.4)	5.0 (4.3 to 5.7)	4.9 (4.1 to 5.8)	5.4 (4.4 to 6.3)	<.001	−2.9 (−4.8 to −1.1)
≥25	4.9 (3.7 to 6.1)	2.9 (1.8 to 4.0)	4.4 (3.0 to 5.7)	4.2 (3.0 to 5.4)	3.4 (2.2 to 4.7)	1.9 (1.4 to 2.3)	2.1 (1.3 to 2.9)	1.2 (0.4 to 2.0)	1.1 (0.8 to 1.5)	0.9 (0.4 to 1.4)	<.001	−4.0 (−5.3 to −2.7)
Alcohol consumption^c^												
None	29.5 (26.2 to 32.8)	31.2 (23.7 to 38.7)	30.1 (25.4 to 34.7)	28.4 (25.2 to 31.7)	28.8 (24.5 to 33.1)	25.1 (22.9 to 27.3)	24.6 (21.3 to 27.9)	26.5 (21.3 to 31.7)	26.6 (23.8 to 29.4)	22.4 (21.1 to 23.6)	<.001	−7.1 (−10.7 to −3.6)
Light	44.6 (43.0 to 46.3)	44.2 (39.3 to 49.2)	45.0 (42.3 to 47.7)	44.5 (41.9 to 47.1)	45.1 (41.9 to 48.3)	44.0 (42.0 to 46.0)	45.2 (42.3 to 48.2)	47.1 (43.6 to 50.7)	47.6 (44.2 to 50.9)	52.2 (50.5 to 54.0)	<.001	7.6 (5.2 to 10.0)
Moderate	16.9 (15.0 to 18.8)	16.5 (13.9 to 19.1)	17.2 (15.0 to 19.3)	17.8 (15.6 to 20.0)	17.9 (15.7 to 20.1)	21.6 (19.6 to 23.6)	19.7 (16.9 to 22.4)	17.8 (16.1 to 19.5)	17.4 (15.4 to 19.5)	17.8 (15.9 to 19.6)	.33	0.9 (−1.8 to 3.6)
Heavy	9.0 (7.4 to 10.6)	8.1 (6.7 to 9.5)	7.7 (6.3 to 9.1)	9.3 (7.8 to 10.8)	8.2 (6.5 to 9.8)	9.3 (7.8 to 10.8)	10.5 (8.8 to 12.2)	8.6 (7.4 to 9.8)	8.4 (6.9 to 9.9)	7.6 (6.6 to 8.6)	.52	−1.4 (−3.3 to 0.5)
Healthy Eating Index–2015 score												
<40	27.2 (23.4 to 31.0)	24.2 (21.9 to 26.5)	24.2 (21.4 to 27.0)	23.0 (21.3 to 24.7)	24.4 (21.3 to 27.5)	21.1 (19.4 to 22.7)	20.4 (18.1 to 22.6)	22.5 (20.6 to 24.3)	23.4 (20.8 to 26.0)	25.8 (23.5 to 28.2)	.59	−1.4 (−5.9 to 3.1)
40-49.9	29.4 (28.0 to 30.8)	28.8 (26.7 to 30.8)	29.8 (27.6 to 32.0)	29.7 (27.3 to 32.2)	26.5 (23.8 to 29.2)	25.5 (23.6 to 27.4)	25.7 (23.8 to 27.7)	26.1 (24.4 to 27.7)	25.1 (23.0 to 27.2)	27.0 (25.2 to 28.7)	<.001	−2.5 (−4.7 to −0.2)
50-59.9	24.1 (21.9 to 26.3)	25.6 (23.9 to 27.4)	26.2 (24.2 to 28.1)	25.4 (23.4 to 27.4)	25.7 (23.8 to 27.6)	26.3 (24.2 to 28.4)	25.0 (23.1 to 26.8)	24.1 (22.9 to 25.4)	24.6 (23.2 to 26.0)	22.7 (20.7 to 24.7)	.03	−1.4 (−4.3 to 1.6)
≥60	19.3 (16.0 to 22.6)	21.4 (18.9 to 23.9)	19.8 (18.1 to 21.5)	21.8 (19.8 to 23.9)	23.4 (18.9 to 27.9)	27.2 (25.6 to 28.8)	29.0 (25.8 to 32.2)	27.3 (25.1 to 29.5)	26.9 (23.6 to 30.1)	24.5 (21.5 to 27.5)	<.001	5.2 (0.8 to 9.7)
Physical activity, min/wk												
0	20.5 (16.8 to 24.1)	15.8 (13.9 to 17.7)	14.1 (12.1 to 16.1)	11.9 (10.1 to 13.7)	22.7 (19.9 to 25.4)	22.1 (19.9 to 24.4)	20.6 (17.9 to 23.3)	23.3 (22.3 to 24.4)	20.3 (17.2 to 23.5)	19.9 (18.4 to 21.4)	<.001	−0.5 (−4.5 to 3.4)
0.1-149.9	23.8 (21.9 to 25.7)	24.6 (22.6 to 26.7)	27.8 (25.1 to 30.4)	25.8 (23.9 to 27.6)	13.0 (11.6 to 14.4)	14.1 (12.2 to 16.0)	13.6 (12.2 to 15.1)	14.0 (12.9 to 15.1)	12.9 (11.6 to 14.2)	11.0 (10.0 to 11.9)	<.001	−12.8 (−15.0 to −10.7)
150.0-300.0	15.4 (13.0 to 17.7)	15.1 (13.7 to 16.5)	17.4 (15.7 to 19.1)	16.2 (14.5 to 17.8)	12.6 (11.1 to 14.0)	13.1 (11.8 to 14.4)	13.1 (11.8 to 14.4)	12.7 (11.2 to 14.3)	11.9 (10.0 to 13.8)	11.1 (9.9 to 12.3)	<.001	−4.2 (−6.9 to −1.6)
>300.0	40.4 (36.6 to 44.1)	44.4 (40.9 to 48.0)	40.8 (37.1 to 44.4)	46.2 (43.0 to 49.4)	51.8 (49.0 to 54.6)	50.7 (46.8 to 54.5)	52.7 (49.7 to 55.6)	49.9 (47.5 to 52.3)	54.9 (52.3 to 57.4)	58.0 (56.2 to 59.8)	<.001	17.6 (13.4 to 21.8)
BMI												
<18.5	2.2 (1.4 to 3.0)	1.9 (1.4 to 2.4)	1.7 (1.2 to 2.1)	1.6 (1.1 to 2.1)	1.5 (0.8 to 2.2)	1.7 (1.1 to 2.2)	1.4 (0.9 to 1.9)	1.5 (1.0 to 2.0)	1.2 (0.8 to 1.7)	1.4 (1.0 to 1.9)	.03	−0.8 (−1.7 to 0.1)
18.5-24.9	33.1 (30.5 to 35.6)	32.5 (30.5 to 34.5)	32.1 (30.4 to 33.9)	30.9 (27.6 to 34.1)	29.9 (28.1 to 31.7)	28.3 (25.5 to 31.1)	29.9 (26.4 to 33.4)	28.0 (26.4 to 29.5)	26.0 (23.5 to 28.6)	24.6 (22.6 to 26.7)	<.001	−8.4 (−11.8 to −5.1)
25.0-29.9	34.4 (31.7 to 37.1)	34.8 (32.3 to 37.3)	33.6 (31.6 to 35.5)	33.5 (31.2 to 35.9)	34.2 (32.6 to 35.7)	33.2 (30.7 to 35.8)	33.5 (30.3 to 36.7)	32.8 (30.9 to 34.6)	33.1 (31.5 to 34.6)	31.7 (29.9 to 33.5)	.03	−2.7 (−5.9 to 0.6)
30.0-34.9	17.4 (15.5 to 19.3)	18.1 (16.3 to 19.9)	20.3 (18.6 to 22.0)	19.3 (17.4 to 21.1)	20.4 (18.9 to 21.8)	20.6 (18.9 to 22.2)	20.6 (18.7 to 22.6)	20.5 (19.1 to 21.9)	21.7 (19.4 to 24.1)	22.4 (20.3 to 24.4)	<.001	5.0 (2.2 to 7.8)
35.0-39.9	8.6 (7.6 to 9.5)	7.4 (6.1 to 8.6)	7.4 (6.5 to 8.3)	8.6 (7.0 to 10.2)	8.3 (7.3 to 9.3)	9.7 (8.7 to 10.7)	7.9 (7.2 to 8.7)	9.4 (8.3 to 10.4)	10.5 (9.1 to 11.9)	10.9 (10.1 to 11.8)	<.001	2.3 (1.1 to 3.6)
≥40.0	4.4 (3.3 to 5.5)	5.3 (4.2 to 6.4)	5.0 (4.1 to 5.9)	6.1 (4.8 to 7.5)	5.7 (4.9 to 6.6)	6.6 (5.8 to 7.3)	6.6 (5.2 to 7.9)	7.8 (6.5 to 9.2)	7.4 (6.2 to 8.7)	8.9 (7.8 to 10.1)	<.001	4.6 (2.9 to 6.2)

Abbreviation: BMI, body mass index (calculated as weight in kilograms divided by height in meters squared).

^a^ Estimated prevalence was adjusted for NHANES survey weights.

^b^ Values may not equal the difference between the first cycle and last cycle estimates because of rounding.

^c^ Light indicates no more than 3 drinks/wk; moderate, more than 3 to 7 drinks/wk for women or more than 3 to 14 drinks/wk for men; and heavy, more than 7 drinks/wk for women or more than 4 drinks/wk for men. For the questionnaire used for the 2017 to March 2020 cycle, use of a list of alcohol use categories vs a continuous scale may affect estimated prevalence across different categories of alcohol consumption.

### Trends in Overall Lifestyle

From the 1999-2000 to the 2017 to March 2020 cycles, improvements were observed in overall lifestyle (Figure 1B). The estimated prevalence significantly increased for healthy lifestyle (≥4 healthy lifestyle factors, from 15.7% to 20.3%; difference, 4.5% [95% CI, 0.7%-8.4%]) and 3 healthy lifestyle factors; and the estimated prevalence significantly decreased for 2, 1, and 0 healthy lifestyle factors (all *P* < .05 for trend). The trend in healthy lifestyle was largely consistent after the adjustment for age or all sociodemographic characteristics (eTable 6 in Supplement 1).

### Trends in Population Subgroups

**Figure 2.**
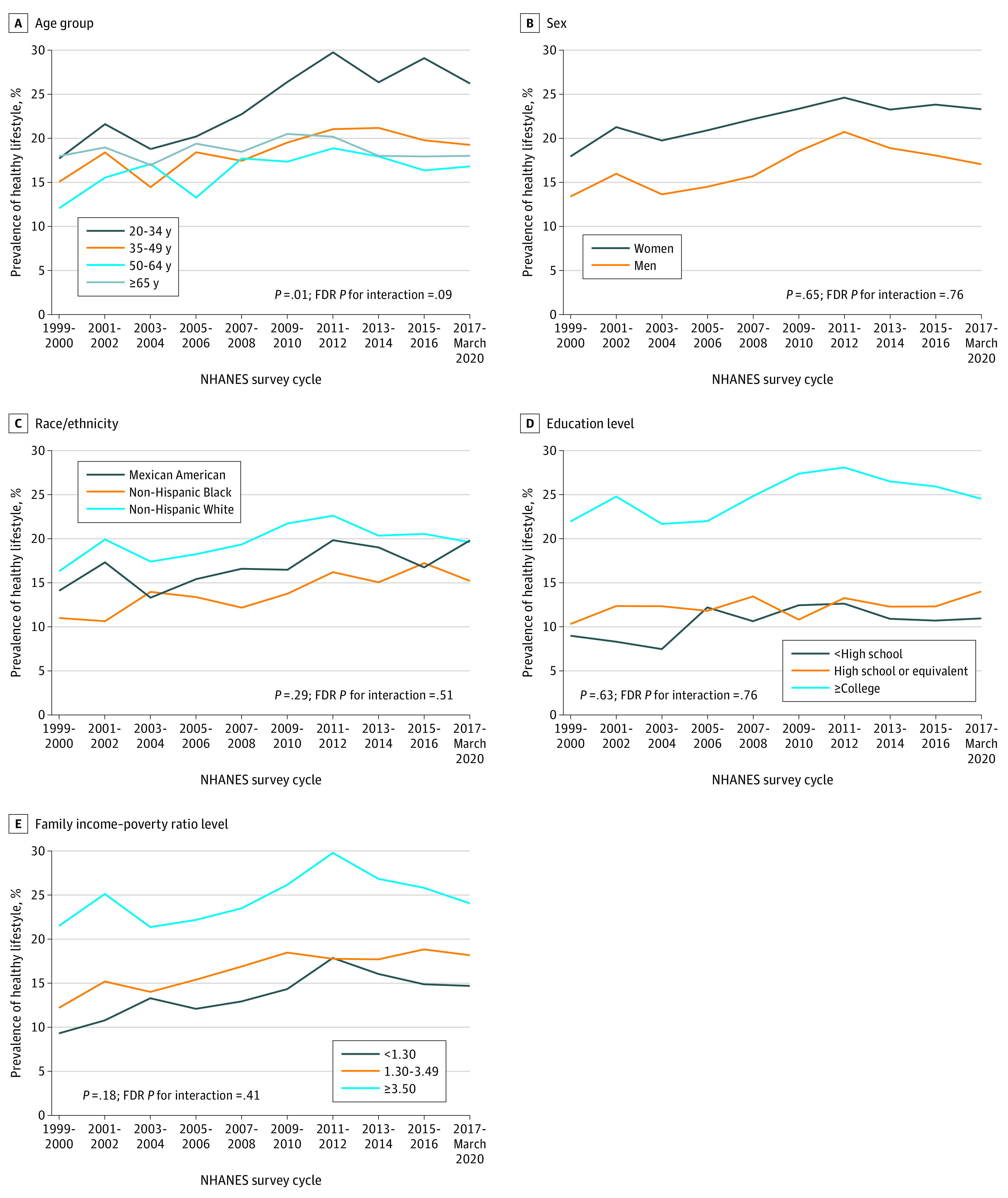
Crude Trends in Estimated Prevalence of Healthy Lifestyle by Age Group, Sex, Race and Ethnicity, Educational Level, and Income, 1999 to March 2020 Heathy lifestyle was defined as 4 or 5 healthy lifestyle factors. Healthy lifestyle factors included never smoking, moderate or lighter alcohol consumption (≤14 drinks/wk for men or ≤7 drinks/wk for women), healthy diet (a Healthy Eating Index–2015 score ≥60.0), sufficient physical activity (≥150 min/wk moderate to vigorous intensity physical activity), and healthy weight (body mass index [calculated as weight in kilograms divided by height in meters squared] 18.5-24.9). Data were adjusted for National Health and Nutrition Examination Survey (NHANES) survey weights. FDR indicates false discovery rate.

Trends in healthy lifestyle across subgroups are shown in Figure 2 and eTable 7 in Supplement 1. From the 1999-2000 to the 2017 to March 2020 cycles, a greater change in healthy lifestyle was observed among younger vs older adults. The estimated prevalence of healthy lifestyle increased among young adults aged 20 to 34 years (difference, 8.5% [95% CI, 3.0%-14.0%]) but was stable among adults 65 years or older (difference, 0.04% [95% CI, −4.28% to 4.35%]). Disparities by race and ethnicity, educational level, and income level were persistent over time (all *P* > .05 and FDR *P* > .05 for interaction), with consistently lower prevalence among non-Hispanic Black adults and adults with low income and educational levels. In addition, there were no significant trends among groups with relatively high prevalence, including non-Hispanic White adults and adults with the highest income level. Trends in individual healthy lifestyle factors across subgroups appear in eTables 8 to 13 in Supplement 1. Among adults 65 years or older, the estimated prevalence of never smoking and healthy diet was not significantly changed, and with a significant decrease in healthy weight. Additionally, greater decreases in healthy weight were observed among Mexican American than non-Hispanic White and non-Hispanic Black adults (FDR *P* = .047 for interaction).

## Discussion

From the 1999-2000 to the 2017 to March 2020 cycles, increases were observed in never smoking, healthy diet, and sufficient physical activity, but a decrease in healthy weight was also observed. Meanwhile, there was no significant change in moderate or lighter alcohol consumption. An improvement in healthy lifestyle was identified, with widening disparity by age group and persistent disparities by race and ethnicity, educational level, and income level.

The tremendous gain in reducing smoking was reflected by an increase in never smoking and decreases in heavy smoking (≥15 cigarettes/d) in our study, which is broadly consistent with declines in current smoking from 1965 to 2020 in previous studies.^7,41^ These improvements may be attributable to the implementation of a series of actions to control tobacco since 1965.^42^ The shifting from no drinking to light drinking in our results is consistent with a recent meta-analysis, which reported increasing alcohol consumption from 2000 to 2016.^9^ For trends in heavy drinking, significant change was observed in 2 previous studies^43,44^ based on different periods (1999-2014 and 2005-2012) and measurement methods (24-hour dietary recalls and consumption in the past month). However, similar to the present findings, both studies indicated relatively low prevalence and small net change. The increase in healthy diet is consistent with prior reports^11,22^ that observed improvements in the American Heart Association diet score from 1999 to 2012 and the mean of HEI-2015 during 1999 to 2016. However, the improvement in healthy diet mainly accumulated before the 2013-2014 cycle; the exact reasons were unclear, and future studies are needed. In a diverse population, physical activity domains other than the leisure-time domain (ie, occupation, household, and transportation) could be important sources of physical activity level.^45^ Hence, we built on and extended a previous study (2007-2016) by assessing trends in combined physical activity from different domains over a 22-year period^12^ and observed an increase in sufficient physical activity. Although the increase in obesity appeared to slow down or level off during 2003 to 2012,^46^ studies covering more recent years (2007-2016) or a longer period (1999-2018) still observed a significant increase.^14,15^ In addition, over the period 1976 to 2006, the overall BMI distribution shifted toward higher BMI.^47^ We observed a transition from healthy weight and overweight to obesity from the 1999-2000 to the 2017 to March 2020 cycles, which may suggest that the increasing shift pattern in BMI still exists. To the best of our knowledge, the present study provides the most comprehensive evaluation of trends in lifestyle factors in the past 22 years.

The increase in healthy lifestyle during the 22-year study period was not consistent with the trends reported between 1988 and 2007, when the prevalence of healthy lifestyle showed significant declines or little net change.^16,17,18^ The inconsistency might be due to relatively higher prevalence of healthy lifestyle prior to 2000, which was mainly driven by lower obesity prevalence and a healthier dietary pattern.^48,49^ During the past 22 years, the improvement in lifestyle is likely to contribute to downward trends in multiple NCDs, such as hypertension (1999-2016),^50^ high cholesterol level (1999-2018),^51^ and coronary heart disease (2001-2012).^52^ However, no studies have observed declines in diabetes,^53,54^ which may be caused by the dominant role of BMI in the risk of diabetes among 5 lifestyle factors.^2^

Our study observed widened disparity in healthy lifestyle by age group with relatively stable prevalence in healthy lifestyle among adults 65 years and older. The smaller improvement in lifestyle among old adults was reflected in no significant change in never smoking and healthy diet with a decrease in healthy weight. Weight gain among old adults was associated with higher all-cause mortality in 2 meta-analyses,^55,56^ and further studies are still needed to explain the weight gain among old adults given that skeletal muscle declines already happen in old adults.^57^ Disparities in life expectancy based on race and ethnicity and socioeconomic status are widespread in the US.^58,59^ Robust disparity in healthy lifestyle by race and ethnicity and socioeconomic levels may partly explain the healthy inequalities.^60^ The nonsignificant improvement in healthy lifestyle among groups with relatively high prevalence is worth concern, since stagnation or reversals of gains in life expectancy among non-Hispanic White adults have been observed from 2000 to 2019.^58^

### Strengths and Limitations

This study’s strengths included use of a series of nationally representative surveys with standardized data collection and rigorous quality control, an updated portrait of lifestyle profiles among US adults, and investigation of differences in trends by key population subgroups. Nevertheless, this study has several limitations. First, data about smoking, alcohol consumption, diet, and physical activity were self-reported and subjected to recall bias. However, the bias might be minimized through trained interviewers and computer-assisted personal interview systems. Second, only 1 valid dietary recall was used in our main analyses. However, we further evaluated the trend in diet quality using 2 dietary recalls and observed similar results. Third, as described, the physical activity questionnaire had changed to Global Physical Activity Questionnaire since the 2007-2008 cycle. However, we used sufficient physical activity to construct the healthy lifestyle score, and the consistent increases in sufficient physical activity for the 1999-2006 and the 2007 to March 2020 cycles provide some support for a modest effect of this change. Fourth, the lifestyle score was derived from the number of healthy lifestyle factors, which may not reflect the unequal effect of individual healthy lifestyle factors.^33^ Fifth, response rates for the NHANES decreased over time.^21^ Nevertheless, sample weights were already included in our analyses according to the NHANES analysis protocol.^37^

## Conclusions

In this cross-sectional study from 1999 to March 2020, we observed different change patterns across 5 healthy lifestyle factors and a modest improvement in overall lifestyle among US adults. Medical care alone is not enough to improve health overall^61^; preventive care is an indispensable component. Changes in food, physical, and policy environments are still needed to improve lifestyle, with attention on old adults and persistent disparity in healthy lifestyle by race and ethnicity and socioeconomic levels. Future studies are warranted to validate our results using other US national surveys.

## Notes

Supplement 1. **eTable 1.** Healthy Eating Index–2015 Components, Component Points, and Scoring Standards **eTable 2.** Number of Participants According to the Number of Healthy Lifestyle Factors Among US Adults 20 Years or Older, 1999 to March 2020 **eTable 3.** Crude Trends in Estimated Prevalence of Healthy Eating Index–2015 Among US Adults 20 Years or Older, 2003 to March 2020 **eTable 4.** Crude Trends in Estimated Prevalence of Physical Activity Among US Adults 20 Years or Older, 1999 to 2006 and 2007 to March 2020 **eTable 5.** Adjusted Trends in Estimated Prevalence of Healthy Lifestyle Factors Among US Adults 20 Years or Older, 1999 to March 2020 **eTable 6.** Adjusted Trends in Estimated Prevalence of Healthy Lifestyle Among US Adults 20 Years or Older, 1999 to March 2020 **eTable 7.** Crude Trends in Estimated Prevalence of Healthy Lifestyle by Age Group, Sex, Race and Ethnicity, Educational Level, and Income, 1999 to March 2020 **eTable 8.** Crude Trends in Estimated Prevalence of Never Smoking by Age Group, Sex, Race and Ethnicity, Educational Level, and Income, 1999 to March 2020 **eTable 9.** Crude Trends in Estimated Prevalence of Moderate or Lighter Alcohol Consumption by Age Group, Sex, Race and Ethnicity, Educational Level, and Income, 1999 to March 2020 **eTable 10.** Crude Trends in Estimated Prevalence of Healthy Diet by Age Group, Sex, Race and Ethnicity, Educational Level, and Income, 1999 to March 2020 **eTable 11.** Crude Trends in Estimated Prevalence of Sufficient Physical Activity by Age Group, Sex, Race and Ethnicity, Educational Level, and Income, 1999 to 2006 **eTable 12.** Crude Trends in Estimated Prevalence of Sufficient Physical Activity by Age Group, Sex, Race and Ethnicity, Educational Level, and Income, 2007 to March 2020 **eTable 13.** Crude Trends in Estimated Prevalence of Healthy Weight by Age Group, Sex, Race and Ethnicity, Educational Level, and Income, 1999 to March 2020

Supplement 2.
Data Sharing Statement
