# Supplementary material for: Trends in Self-Reported Adherence to Healthy Lifestyle Behaviors Among US Adults, 1999 to March 2020
Source: JAMA Netw Open. 2023 Jul 14;6(7):e2323584. doi: 10.1001/jamanetworkopen.2023.23584 (PMC10349344; doi:10.1001/jamanetworkopen.2023.23584)
Supplement: Supplement 1. — eTable 1. Healthy Eating Index–2015 Components, Component Points, and Scoring Standards eTable 2. Number of Participants According to the Number of Healthy Lifestyle Factors Among US Adults 20 Years or Older, 1999 to March 2020 eTable 3. Crude Trends in Estimated Prevalence of Healthy Eating Index–2015 Among US Adults 20 Years or Older, 2003 to March 2020 eTable 4. Crude Trends in Estimated Prevalence of Physical Activity Among US Adults 20 Years or Older, 1999 to 2006 and 2007 to March 2020 eTable 5. Adjusted Trends in Estimated Prevalence of Healthy Lifestyle Factors Among US Adults 20 Years or Older, 1999 to March 2020 eTable 6. Adjusted Trends in Estimated Prevalence of Healthy Lifestyle Among US Adults 20 Years or Older, 1999 to March 2020 eTable 7. Crude Trends in Estimated Prevalence of Healthy Lifestyle by Age Group, Sex, Race and Ethnicity, Educational Level, and Income, 1999 to March 2020 eTable 8. Crude Trends in Estimated Prevalence of Never Smoking by Age Group, Sex, Race and Ethnicity, Educational Level, and Income, 1999 to March 2020 eTable 9. Crude Trends in Estimated Prevalence of Moderate or Lighter Alcohol Consumption by Age Group, Sex, Race and Ethnicity, Educational Level, and Income, 1999 to March 2020 eTable 10. Crude Trends in Estimated Prevalence of Healthy Diet by Age Group, Sex, Race and Ethnicity, Educational Level, and Income, 1999 to March 2020 eTable 11. Crude Trends in Estimated Prevalence of Sufficient Physical Activity by Age Group, Sex, Race and Ethnicity, Educational Level, and Income, 1999 to 2006 eTable 12. Crude Trends in Estimated Prevalence of Sufficient Physical Activity by Age Group, Sex, Race and Ethnicity, Educational Level, and Income, 2007 to March 2020 eTable 13. Crude Trends in Estimated Prevalence of Healthy Weight by Age Group, Sex, Race and Ethnicity, Educational Level, and Income, 1999 to March 2020 [file jamanetwopen-e2323584-s001.pdf]

Supplementary Online Content

Li Y, Xia PF, Geng TT, et al. Trends in self-reported adherence to healthy lifestyle behaviors among US adults, 1999 to March 2020. *JAMA Netw Open*. 2023;6(7):e2323584. doi:10.1001/jamanetworkopen.2023.23584

eTable 1. Healthy Eating Index–2015 Components, Component Points, and Scoring Standards
eTable 2. Number of Participants According to the Number of Healthy Lifestyle Factors Among US Adults 20 Years or Older, 1999 to March 2020
eTable 3. Crude Trends in Estimated Prevalence of Healthy Eating Index–2015 Among US Adults 20 Years or Older, 2003 to March 2020
eTable 4. Crude Trends in Estimated Prevalence of Physical Activity Among US Adults 20 Years or Older, 1999 to 2006 and 2007 to March 2020
eTable 5. Adjusted Trends in Estimated Prevalence of Healthy Lifestyle Factors Among US Adults 20 Years or Older, 1999 to March 2020
eTable 6. Adjusted Trends in Estimated Prevalence of Healthy Lifestyle Among US Adults 20 Years or Older, 1999 to March 2020
eTable 7. Crude Trends in Estimated Prevalence of Healthy Lifestyle by Age Group, Sex, Race and Ethnicity, Educational Level, and Income, 1999 to March 2020
eTable 8. Crude Trends in Estimated Prevalence of Never Smoking by Age Group, Sex, Race and Ethnicity, Educational Level, and Income, 1999 to March 2020
eTable 9. Crude Trends in Estimated Prevalence of Moderate or Lighter Alcohol Consumption by Age Group, Sex, Race and Ethnicity, Educational Level, and Income, 1999 to March 2020
eTable 10. Crude Trends in Estimated Prevalence of Healthy Diet by Age Group, Sex, Race and Ethnicity, Educational Level, and Income, 1999 to March 2020
eTable 11. Crude Trends in Estimated Prevalence of Sufficient Physical Activity by Age Group, Sex, Race and Ethnicity, Educational Level, and Income, 1999 to 2006
eTable 12. Crude Trends in Estimated Prevalence of Sufficient Physical Activity by Age Group, Sex, Race and Ethnicity, Educational Level, and Income, 2007 to March 2020
eTable 13. Crude Trends in Estimated Prevalence of Healthy Weight by Age Group, Sex, Race and Ethnicity, Educational Level, and Income, 1999 to March 2020

This supplementary material has been provided by the authors to give readers additional information about their work.

© 2023 Li Y et al. *JAMA Network Open*.

eTable 1. Healthy Eating Index–2015 Components, Component Points, and Scoring Standards[a]

Component	Maximum points	Standard for maximum score	Standard for minimum score of zero
**Adequacy**			
Total Fruits[b]	5	≥0.8 cup equiv. per 1,000 kcal	No Fruit
Whole Fruits[c]	5	≥0.4 cup equiv. per 1,000 kcal	No Whole Fruit
Total Vegetables[d]	5	≥1.1 cup equiv. per 1,000 kcal	No Vegetables
Greens and Beans	5	≥0.2 cup equiv. per 1,000 kcal	No Dark Green Vegetables or Legumes
Whole Grains	10	≥1.5 oz equiv. per 1,000 kcal	No Whole Grains
Dairy[e]	10	≥1.3 cup equiv. per 1,000 kcal	No Dairy
Total Protein Foods[f]	5	≥2.5 oz equiv. per 1,000 kcal	No Protein Foods
Seafood and Plant Proteins[f,g]	5	≥0.8 oz equiv. per 1,000 kcal	No Seafood or Plant Proteins
Fatty Acids[h]	10	(PUFAs + MUFAs)/SFAs ≥2.5	(PUFAs + MUFAs)/SFAs ≤1.2
**Moderation**			
Refined Grains	10	≤1.8 oz equiv. per 1,000 kcal	≥4.3 oz equiv. per 1,000 kcal
Sodium	10	≤1.1 gram per 1,000 kcal	≥2.0 grams per 1,000 kcal
Added Sugars	10	≤6.5% of energy	≥26% of energy
Saturated Fats	10	≤8% of energy	≥16% of energy

[a] Intakes between the minimum and maximum standards are scored proportionately.

[b] Includes 100% fruit juice.

[c] Includes all forms except juice.

[d] Includes legumes (beans and peas).

[e] Includes all milk products, such as fluid milk, yogurt, and cheese, and fortified soy beverages.

[f] Includes legumes (beans and peas).

[g] Includes seafood, nuts, seeds, soy products (other than beverages), and legumes (beans and peas).

[h] Ratio of poly- and monounsaturated fatty acids (PUFAs and MUFAs) to saturated fatty acids (SFAs).

© 2023 Li Y et al. *JAMA Network Open*.

eTable 2. Number of Participants According to the Number of Healthy Lifestyle Factors Among US Adults 20 Years or Older, 1999 to March 2020[a]

Number of healthy lifestyle factors	Number of participants
**Zero to five**	
1999-2000	3967
2001-2002	4295
2003-2004	4120
2005-2006	4184
2007-2008	5001
2009-2010	5196
2011-2012	4372
2013-2014	4740
2015-2016	4652
2017-March 2020	7325
**Five**	
1999-2000	96
2001-2002	123
2003-2004	94
2005-2006	112
2007-2008	114
2009-2010	150
2011-2012	182
2013-2014	212
2015-2016	171
2017-March 2020	221

[a] Healthy lifestyle factors included never smoking, non-heavy drinking (≤14 drinks/week for men or ≤7 drinks/week for women), healthy diet (a Healthy Eating Index-2015 score ≥60), sufficient physical activity (≥150 minutes/week moderate to vigorous intensity physical activity), normal weight (body mass index at 18.5–24.9 kg/m^2).

© 2023 Li Y et al. *JAMA Network Open*.

eTable 3. Crude Trends in Estimated Prevalence of Healthy Eating Index–2015 Among US Adults 20 Years or Older, 2003 to March 2020

	Estimated prevalence of Healthy Eating Index-2015, % (95% CI)[a]									
	**2003-2004 (n = 3835)**	**2005-2006 (n = 3790)**	**2007-2008 (n = 4365)**	**2009-2010 (n = 4583)**	**2011-2012 (n = 3940)**	**2013-2014 (n = 4235)**	**2015-2016 (n = 3933)**	**2017-March 2020 (n =6349)**	***P* value for trend**	**Difference, 2017-March 2020 vs 2003-2004 (95% CI)[b]**
**Healthy Eating Index-2015 score**										
<40	17.8 (15.4-20.3)	15.5 (13.2-17.7)	16.2 (14.1-18.3)	13.7 (12.4-15.0)	13.6 (12.3-14.9)	15.1 (14.0-16.1)	16.3 (13.3-19.2)	17.6 (15.1-20.0)	.60	-0.3 (-3.7 to 3.2)
40-49.9	29.3 (27.1-31.5)	29.2 (27.1-31.2)	27.9 (24.3-31.4)	25.0 (23.2-26.8)	23.3 (20.7-25.9)	25.9 (23.8-28.0)	25.1 (22.4-27.8)	25.5 (23.1-27.9)	.005	-3.8 (-7.1 to -0.5)
50-59.9	29.1 (27.6-30.6)	27.6 (25.6-29.6)	26.2 (24.7-27.7)	29.1 (26.8-31.3)	28.0 (25.8-30.2)	24.5 (22.9-26.2)	26.3 (24.3-28.4)	27.4 (25.3-29.6)	.18	-1.6 (-4.3 to 1.0)
≥60	23.8 (20.8-26.8)	27.8 (25.3-30.2)	29.7 (25.1-34.4)	32.2 (30.5-34.0)	35.1 (31.8-38.3)	34.5 (32.2-36.8)	32.2 (27.8-36.7)	29.5 (26.8-32.2)	.007	5.7 (1.7 to 9.8)

[a] Estimated prevalence were adjusted for NHANES survey weights.

[b] Values may not equal the difference between the first cycle and last cycle estimates because of rounding.

© 2023 Li Y et al. *JAMA Network Open*.

eTable 4. Crude Trends in Estimated Prevalence of Physical Activity Among US Adults 20 Years or Older, 1999 to 2006 and 2007 to March 2020

	**Estimated prevalence of physical activity (95% CI)[a]**						**Estimated prevalence of physical activity (95% CI)[a]**							
	**1999-2000 (n = 3967)**	**2001-2002 (n = 4295)**	**2003-2004 (n = 4120)**	**2005-2006 (n = 4184)**	***P* value for trend**	**Difference, 2005-2006 vs 1999-2000 (95% CI)[b]**	**2007-2008 (n = 5001)**	**2009-2010 (n = 5196)**	**2011-2012 (n = 4372)**	**2013-2014 (n = 4740)**	**2015-2016 (n = 4652)**	**2017-March 2020 (n = 7325)**	***P* value for trend**	**Difference, 2017-March 2020 vs 2007-2008 (95% CI)[b]**
**Physical activity, minutes/week**														
0	20.5 (16.8-24.1)	15.8 (13.9-17.7)	14.1 (12.1-16.1)	11.9 (10.1-13.7)	<.001	-8.6 (-12.7 to -4.5)	22.7 (19.9-25.4)	22.1 (19.9-24.4)	20.6 (17.9-23.3)	23.3 (22.3-24.4)	20.3 (17.2-23.5)	19.9 (18.4-21.4)	.06	-2.7 (-5.9 to 0.4)
0.1-149.9	23.8 (21.9-25.7)	24.6 (22.6-26.7)	27.8 (25.1-30.4)	25.8 (23.9-27.6)	.048	1.9 (-0.7 to 4.6)	13.0 (11.6-14.4)	14.1 (12.2-16.0)	13.6 (12.2-15.1)	14.0 (12.9-15.1)	12.9 (11.6-14.2)	11.0 (10.0-11.9)	.002	-2.0 (-3.7 to -0.3)
150-300	15.4 (13.0-17.7)	15.1 (13.7-16.5)	17.4 (15.7-19.1)	16.2 (14.5-17.8)	.31	0.8 (-2.2 to 3.8)	12.6 (11.1-14.0)	13.1 (11.8-14.4)	13.1 (11.8-14.4)	12.7 (11.2-14.3)	11.9 (10.0-13.8)	11.1 (9.9-12.3)	.03	-1.5 (-3.3 to 0.4)
>300	40.4 (36.6-44.1)	44.4 (40.9-48.0)	40.8 (37.1-44.4)	46.2 (43.0-49.4)	.09	5.9 (0.8 to 10.9)	51.8 (49.0-54.6)	50.7 (46.8-54.5)	52.7 (49.7-55.6)	49.9 (47.5-52.3)	54.9 (52.3-57.4)	58.0 (56.2-59.8)	<.001	6.2 (2.9 to 9.5)
≥150	55.7 (51.8-59.6)	59.6 (56.3-62.8)	58.1 (54.8-61.5)	62.4 (59.3-65.5)	.02	6.7 (1.6 to 11.7)	64.4 (61.1-67.6)	63.8 (60.5-67.1)	65.8 (63.1-68.5)	62.6 (61.0-64.2)	66.7 (63.3-70.2)	69.1 (67.2-71.1)	.003	4.8 (0.9 to 8.6)

[a] Estimated prevalence were adjusted for NHANES survey weights.

[b] Values may not equal the difference between the first cycle and last cycle estimates because of rounding.

© 2023 Li Y et al. *JAMA Network Open*.

eTable 5. Adjusted Trends in Estimated Prevalence of Healthy Lifestyle Factors Among US Adults 20 Years or Older, 1999 to March 2020[a]

	Estimated prevalence of healthy lifestyle factors, % (95% CI)	
	Age-adjusted	Multivariable-adjusted[b]
**Never smoking**		
1999-2000	48.5 (45.5 to 51.5)	52.4 (49.7-55.0)
2001-2002	50.2 (45.8 to 54.7)	53.1 (48.7-57.6)
2003-2004	47.6 (45.9 to 49.4)	50.6 (48.9-52.3)
2005-2006	50.0 (48.1 to 51.9)	52.3 (50.1-54.6)
2007-2008	51.3 (48.1 to 54.5)	53.8 (51.5-56.0)
2009-2010	54.6 (51.0 to 58.2)	56.9 (53.8-60.0)
2011-2012	54.4 (51.5 to 57.2)	56.1 (53.4-58.7)
2013-2014	56.4 (53.3 to 59.4)	57.8 (55.0-60.6)
2015-2016	55.5 (53.5 to 57.4)	56.1 (54.4-57.7)
2017-March 2020	57.4 (55.3 to 59.6)	57.6 (55.9-59.4)
P for trend	<.001	<.001
Difference, 2017-March 2020 vs 1999-2000 (95% CI)[c]	8.9 (5.2 to 12.7)	5.3 (2.2 to 8.3)
**Non-heavy drinking**		
1999-2000	91.3 (89.7 to 92.9)	92.9 (91.2-94.5)
2001-2002	92.3 (90.9 to 93.7)	93.9 (92.5-95.3)
2003-2004	92.6 (91.3 to 94.0)	94.2 (92.7-95.7)
2005-2006	91.0 (89.5 to 92.6)	92.6 (91.0-94.1)
2007-2008	92.2 (90.5 to 93.9)	93.7 (92.0-95.4)
2009-2010	91.1 (89.6 to 92.6)	92.4 (91.0-93.9)
2011-2012	89.8 (88.1 to 91.5)	91.0 (89.2-92.8)
2013-2014	91.7 (90.5 to 92.9)	92.8 (91.6-94.0)
2015-2016	91.8 (90.4 to 93.2)	92.8 (91.4-94.1)
2017-March 2020	92.6 (91.5 to 93.6)	93.5 (92.5-94.5)
P for trend	.68	.50
Difference, 2017-March 2020 vs 1999-2000 (95% CI)[c]	1.3 (-0.6 to 3.1)	0.7 (-1.3 to 2.6)
**Healthy diet**		
1999-2000	20.6 (17.4 to 23.8)	20.2 (17.6-22.9)
2001-2002	22.8 (20.3 to 25.3)	21.7 (19.4-24.0)
2003-2004	21.0 (19.3 to 22.6)	20.2 (18.9-21.5)
2005-2006	22.9 (21.1 to 24.7)	21.7 (20.0-23.4)
2007-2008	24.5 (19.9 to 29.1)	23.5 (19.6-27.3)
2009-2010	28.1 (26.3 to 29.9)	26.8 (25.1-28.5)
2011-2012	29.7 (26.5 to 33.0)	28.0 (25.0-30.9)

© 2023 Li Y et al. *JAMA Network Open*.

	Estimated prevalence of healthy lifestyle factors, % (95% CI)	
	Age-adjusted	Multivariable-adjusted[b]
2013-2014	28.1 (25.8 to 30.3)	26.3 (24.5-28.0)
2015-2016	27.3 (24.2 to 30.4)	25.0 (22.2-27.7)
2017-March 2020	25.0 (22.0 to 28.0)	22.6 (19.9-25.2)
P for trend	<.001	.006
Difference, 2017-March 2020 vs 1999-2000 (95% CI)[c]	4.4 (0.0 to 8.7)	2.3 (-1.5 to 6.2)
**Sufficient physical activity**		
1999-2000	54.1 (50.1 to 58.1)	49.4 (45.8-53.0)
2001-2002	57.9 (54.5 to 61.3)	51.7 (48.6-54.8)
2003-2004	56.7 (53.3 to 60.0)	50.6 (47.4-53.8)
2005-2006	61.0 (57.8 to 64.3)	54.6 (51.7-57.4)
2007-2008	62.9 (59.7 to 66.1)	57.4 (54.6-60.1)
2009-2010	62.5 (59.3 to 65.7)	56.6 (53.8-59.4)
2011-2012	64.7 (62.3 to 67.0)	58.4 (56.1-60.8)
2013-2014	61.6 (59.8 to 63.3)	55.4 (53.6-57.2)
2015-2016	66.1 (62.9 to 69.3)	59.6 (57.1-62.1)
2017-March 2020	68.4 (66.5 to 70.3)	61.8 (59.8-63.7)
P for trend	<.001	<.001
Difference, 2017-March 2020 vs 1999-2000 (95% CI)[c]	14.3 (9.9 to 18.7)	12.4 (12.3 to 12.4)
**Normal weight**		
1999-2000	32.4 (29.6 to 35.2)	29.3 (26.7-31.8)
2001-2002	32.1 (30.3 to 34.0)	28.9 (26.9-30.8)
2003-2004	31.6 (29.6 to 33.6)	28.6 (26.6-30.6)
2005-2006	30.5 (27.5 to 33.6)	27.5 (24.4-30.5)
2007-2008	29.6 (27.8 to 31.5)	26.5 (24.7-28.3)
2009-2010	28.1 (25.4 to 30.7)	25.0 (22.4-27.5)
2011-2012	29.6 (26.4 to 32.8)	26.3 (23.3-29.2)
2013-2014	27.7 (26.1 to 29.3)	24.5 (22.9-26.1)
2015-2016	25.8 (23.2 to 28.4)	22.4 (20.0-24.7)
2017-March 2020	24.4 (22.3 to 26.4)	20.9 (19.0-22.8)
P for trend	<.001	<.001
Difference, 2017-March 2020 vs 1999-2000 (95% CI)[c]	-8.0 (-11.5 to -4.5)	-8.3 (-11.7 to -5.0)

[a] Healthy lifestyle factors included never smoking, non-heavy drinking (≤14 drinks/week for men or ≤7 drinks/week for women), healthy diet (a Healthy Eating Index-2015 score ≥60), sufficient physical activity (≥150 minutes/week moderate to vigorous intensity physical activity), normal weight (body mass index at 18.5–24.9 kg/m^2).

© 2023 Li Y et al. *JAMA Network Open*.

[b] Multivariable adjusted models included age group (20-34, 35-49, 50-64, ≥65 y), sex (women, men), race/ethnicity (non-Hispanic White, non-Hispanic Black, Mexican American, other), education level (less than high school, high school or equivalent, college or above), and family income–poverty ratio level (<1.30, 1.30-3.49, ≥3.50). Missing values in education and income (n=38 without education data; n=4065 without income data) were imputed using multiple imputation.

[c] Values may not equal the difference between the first cycle and last cycle estimates because of rounding.

© 2023 Li Y et al. *JAMA Network Open*.

eTable 6. Adjusted Trends in Estimated Prevalence of Healthy Lifestyle Among US Adults 20 Years or Older, 1999 to March 2020[a]

	**Estimated prevalence of healthy lifestyle, % (95% CI)**	
	**Age-adjusted**	**Multivariable-adjusted[b]**
1999-2000	15.4 (12.4 to 18.4)	14.0 (11.7-16.4)
2001-2002	18.5 (16.3 to 20.7)	16.3 (14.1-18.4)
2003-2004	16.5 (14.1 to 19.0)	14.6 (12.5-16.7)
2005-2006	17.7 (15.2 to 20.1)	15.3 (13.0-17.6)
2007-2008	19.0 (15.0 to 23.0)	16.9 (13.7-20.0)
2009-2010	20.9 (17.8 to 23.9)	18.5 (15.6-21.3)
2011-2012	22.6 (18.6 to 26.6)	19.6 (16.0-23.1)
2013-2014	21.0 (18.9 to 23.1)	18.0 (16.3-19.7)
2015-2016	20.9 (18.5 to 23.3)	17.3 (15.4-19.3)
2017-March 2020	20.1 (17.7 to 22.6)	16.5 (14.4-18.5)
P for trend	<.001	.04
Difference, 2017-March 2020 vs 1999-2000 (95% CI)[c]	4.8 (0.8 to 8.7)	2.4 (-0.9 to 5.7)

[a] Healthy lifestyle was defined as 4 or 5 of healthy lifestyle factors.

[b] Multivariable adjusted models included age group (20-34, 35-49, 50-64, ≥65 y), sex (women, men), race/ethnicity (non-Hispanic White, non-Hispanic Black, Mexican American, other), education level (less than high school, high school or equivalent, college or above), and family income–poverty ratio level (<1.30, 1.30-3.49, ≥3.50). Missing values in education and income (n=38 without education data; n=4065 without income data) were imputed using multiple imputation.

[c] Values may not equal the difference between the first cycle and last cycle estimates because of rounding.

© 2023 Li Y et al. *JAMA Network Open*.

eTable 7. Crude Trends in Estimated Prevalence of Healthy Lifestyle by Age Group, Sex, Race and Ethnicity, Educational Level, and Income, 1999 to March 2020[a]

	Estimated prevalence of healthy lifestyle (95% CI)[b]											Difference, 2017-March 2020 vs 1999-2000 (95% CI)[b]
	**1999-2000**	**2001-2002**	**2003-2004**	**2005-2006**	**2007-2008**	**2009-2010**	**2011-2012**	**2013-2014**	**2015-2016**	**2017-March 2020**	***P* value for trend**	
**Age group, y**												
20-34	17.7 (14.3-21.2)	21.6 (14.6-28.6)	18.8 (14.9-22.7)	20.2 (15.6-24.8)	22.7 (17.0-28.4)	26.4 (19.0-33.7)	29.8 (22.1-37.4)	26.4 (22.8-30.0)	29.1 (23.5-34.7)	26.2 (22.0-30.4)	<.001	8.5 (3.0 to 14.0)
35-49	15.1 (9.5-20.6)	18.4 (15.1-21.7)	14.5 (10.5-18.4)	18.4 (15.5-21.3)	17.4 (13.2-21.7)	19.5 (16.1-22.9)	21.0 (16.0-26.1)	21.2 (18.0-24.4)	19.8 (15.9-23.7)	19.3 (15.8-22.7)	.04	4.2 (-2.4 to 10.8)
50-64	12.1 (8.9-15.2)	15.5 (12.7-18.4)	17.1 (13.3-20.8)	13.3 (9.6-17.0)	17.7 (12.7-22.7)	17.3 (13.7-21.0)	18.9 (15.3-22.4)	17.9 (14.9-21.0)	16.4 (13.4-19.3)	16.8 (14.0-19.7)	.07	4.7 (0.5 to 9.0)
≥65	18.0 (15.4-20.5)	18.9 (15.4-22.5)	17.0 (14.1-19.8)	19.4 (14.8-24.0)	18.5 (14.0-22.9)	20.5 (17.2-23.8)	20.2 (15.1-25.2)	18.0 (16.2-19.8)	17.9 (13.9-22.0)	18.0 (14.6-21.4)	.88	0.04 (-4.28 to 4.35)
P value for interaction	.01											
FDR *P* value for interaction	.09											
**Sex**												
Women	17.9 (14.3-21.6)	21.3 (18.7-23.9)	19.8 (16.2-23.4)	20.9 (18.0-23.8)	22.2 (17.2-27.2)	23.4 (20.4-26.3)	24.6 (20.2-29.0)	23.3 (20.8-25.7)	23.8 (20.5-27.2)	23.3 (20.6-26.0)	.004	5.4 (0.8 to 10.0)
Men	13.4 (10.7-	16.0 (13.2-	13.6 (11.8-	14.5 (11.7-	15.7 (12.0-	18.5 (14.7-	20.7 (16.2-	18.9 (16.4-	18.1 (15.3-	17.1 (14.4-	.002	3.7 (-0.2 to 7.5)

© 2023 Li Y et al. *JAMA Network Open*.

	Estimated prevalence of healthy lifestyle (95% CI)[b]											
	**1999-2000**	**2001-2002**	**2003-2004**	**2005-2006**	**2007-2008**	**2009-2010**	**2011-2012**	**2013-2014**	**2015-2016**	**2017-March 2020**	***P* value for trend**	**Difference, 2017-March 2020 vs 1999-2000 (95% CI)[b]**
	16.1)	18.8)	15.5)	17.3)	19.4)	22.3)	25.2)	21.4)	20.8)	19.7)		
P value for interaction						.65						
FDR *P* value for interaction						.76						
**Race/ethnicity**												
Non-Hispanic White	16.3 (12.1-20.5)	19.9 (17.5-22.4)	17.4 (14.7-20.1)	18.2 (15.0-21.5)	19.3 (13.6-25.1)	21.7 (17.5-26.0)	22.6 (17.2-28.0)	20.3 (17.6-23.1)	20.5 (17.9-23.2)	19.6 (16.2-23.0)	.10	3.3 (-2.2 to 8.7)
Non-Hispanic Black	11.0 (7.2-14.9)	10.6 (7.0-14.2)	14.0 (9.4-18.5)	13.4 (10.8-16.0)	12.2 (10.0-14.4)	13.8 (9.8-17.7)	16.2 (13.8-18.6)	15.0 (11.6-18.5)	17.2 (12.9-21.5)	15.2 (13.1-17.3)	.004	4.2 (-0.2 to 8.6)
Mexican American	14.1 (10.1-18.1)	17.3 (13.2-21.4)	13.3 (8.6-18.0)	15.4 (12.1-18.7)	16.6 (13.3-19.9)	16.5 (11.8-21.1)	19.8 (14.6-25.1)	19.0 (15.3-22.7)	16.7 (13.6-19.8)	19.8 (15.9-23.7)	.02	5.7 (0.1 to 11.3)
P value for interaction						.29						
FDR *P* value for interaction						.51						
**Education level**												
Less than high school	9.0 (7.2-10.7)	8.3 (4.5-12.1)	7.5 (5.4-9.5)	12.2 (9.4-15.0)	10.6 (7.6-13.7)	12.4 (9.9-15.0)	12.6 (9.3-16.0)	10.9 (7.9-13.9)	10.7 (7.7-13.7)	10.9 (8.0-13.9)	.03	2.0 (-1.5 to 5.4)
High school or equivalent	10.3 (7.3-13.3)	12.4 (8.7-16.1)	12.3 (9.5-15.2)	11.8 (9.1-14.5)	13.5 (9.4-17.5)	10.8 (8.8-12.8)	13.3 (9.3-17.2)	12.3 (9.8-14.8)	12.3 (9.4-15.2)	14.0 (11.5-16.5)	.16	3.7 (-0.3 to 7.6)

© 2023 Li Y et al. *JAMA Network Open*.

	Estimated prevalence of healthy lifestyle (95% CI)[b]											
	1999-2000	2001-2002	2003-2004	2005-2006	2007-2008	2009-2010	2011-2012	2013-2014	2015-2016	2017-March 2020	*P* value for trend	Difference, 2017-March 2020 vs 1999-2000 (95% CI)[b]
College or above	21.9 (17.7-26.2)	24.8 (22.3-27.3)	21.7 (18.7-24.7)	22.0 (18.3-25.7)	24.8 (20.5-29.1)	27.4 (23.1-31.7)	28.1 (23.3-32.9)	26.5 (23.7-29.2)	25.9 (23.5-28.3)	24.5 (21.9-27.2)	.046	2.6 (-2.5 to 7.6)
P value for interaction					.63							
FDR *P* value for interaction					.76							
**Family income–poverty ratio level[c]**												
<1.30	9.3 (5.0-13.6)	10.8 (6.5-15.0)	13.3 (10.4-16.2)	12.1 (9.3-14.9)	12.9 (8.6-17.2)	14.3 (10.9-17.8)	17.8 (11.0-24.7)	16.0 (12.2-19.9)	14.9 (10.9-18.8)	14.7 (11.6-17.8)	.005	5.4 (0.0 to 10.7)
1.30-3.49	12.2 (9.0-15.4)	15.2 (10.6-19.8)	14.0 (10.5-17.5)	15.4 (12.9-17.9)	16.9 (12.0-21.8)	18.5 (15.8-21.1)	17.8 (14.8-20.7)	17.7 (14.2-21.2)	18.8 (15.5-22.2)	18.2 (15.6-20.7)	.002	6.0 (1.8 to 10.1)
≥3.50	21.5 (16.4-26.6)	25.1 (22.4-27.8)	21.4 (17.9-24.8)	22.2 (18.2-26.1)	23.5 (18.6-28.4)	26.2 (20.3-32.0)	29.8 (23.6-36.0)	26.8 (23.9-29.8)	25.8 (23.6-28.0)	24.1 (20.5-27.6)	.10	2.6 (-3.7 to 8.8)
P value for interaction					.18							
FDR *P* value for interaction					.41							

Abbreviations: FDR, false discovery rate.

[a] Estimated prevalence were adjusted for NHANES survey weights.

[b] Values may not equal the difference between the first cycle and last cycle estimates because of rounding.

[c] The variable was calculated by dividing family income by the poverty guidelines. A higher ratio represents a higher level of income.

© 2023 Li Y et al. *JAMA Network Open*.

eTable 8. Crude Trends in Estimated Prevalence of Never Smoking by Age Group, Sex, Race and Ethnicity, Educational Level, and Income, 1999 to March 2020

	Estimated prevalence of never smoking (95% CI)[a]											Difference, 2017-March 2020 vs 1999-2000 (95% CI)[b]
	**1999-2000**	**2001-2002**	**2003-2004**	**2005-2006**	**2007-2008**	**2009-2010**	**2011-2012**	**2013-2014**	**2015-2016**	**2017-March 2020**	***P* value for trend**	
**Age group, y**												
20-34	56.9 (51.3-62.6)	56.9 (48.4-65.3)	57.6 (53.7-61.6)	53.9 (48.9-58.9)	55.7 (49.1-62.2)	61.4 (54.5-68.2)	63.6 (56.7-70.4)	64.6 (60.6-68.6)	65.2 (60.9-69.5)	66.5 (61.4-71.6)	<.001	9.6 (1.9 to 17.2)
35-49	47.5 (43.1-51.9)	53.5 (47.1-59.8)	46.0 (41.6-50.4)	53.9 (51.6-56.1)	54.2 (49.7-58.7)	56.7 (53.1-60.4)	57.1 (52.3-62.0)	59.1 (55.3-62.9)	58.4 (53.7-63.1)	56.8 (52.1-61.5)	<.001	9.3 (2.8 to 15.8)
50-64	42.3 (37.5-47.2)	40.3 (35.7-44.9)	42.6 (38.9-46.2)	45.7 (41.5-49.9)	47.6 (44.8-50.5)	49.3 (44.4-54.2)	48.8 (44.3-53.3)	50.5 (45.1-55.9)	49.0 (44.3-53.8)	54.5 (49.8-59.1)	<.001	12.1 (5.3 to 18.9)
≥65	49.0 (45.1-52.9)	51.4 (46.8-56.1)	44.8 (40.6-48.9)	46.6 (40.7-52.5)	48.1 (44.4-51.7)	51.1 (48.2-53.9)	46.9 (42.6-51.2)	50.9 (46.7-55.2)	48.7 (44.6-52.8)	51.4 (47.7-55.0)	.20	2.4 (-3.0 to 7.8)
P value for interaction	.01											
FDR *P* value for interaction	.09											
**Sex**												
Women	57.5 (55.4-59.6)	57.7 (52.7-62.6)	55.4 (52.5-58.3)	56.9 (54.2-59.7)	58.8 (55.2-62.5)	60.3 (56.8-63.8)	61.1 (57.0-65.1)	61.7 (58.2-65.1)	63.0 (60.0-66.0)	64.2 (61.5-66.9)	<.001	6.7 (3.3 to 10.2)
Men	40.8 (36.4-	44.0 (38.8-	40.7 (37.5-	43.7 (40.6-	44.0 (40.0-	49.6 (44.9-	48.2 (43.7-	51.6 (48.1-	47.9 (45.2-	50.8 (48.1-	<.001	10.0 (4.8 to 15.2)

© 2023 Li Y et al. *JAMA Network Open*.

	Estimated prevalence of never smoking (95% CI)[a]											
	**1999-2000**	**2001-2002**	**2003-2004**	**2005-2006**	**2007-2008**	**2009-2010**	**2011-2012**	**2013-2014**	**2015-2016**	**2017-March 2020**	***P* value for trend**	**Difference, 2017-March 2020 vs 1999-2000 (95% CI)[b]**
	45.3)	49.2)	43.9)	46.9)	48.1)	54.2)	52.7)	55.1)	50.7)	53.5)		
P value for interaction						.37						
FDR *P* value for interaction						.62						
**Race/ethnicity**												
Non-Hispanic White	46.1 (42.4-49.9)	49.4 (43.9-54.9)	45.4 (43.4-47.4)	47.0 (44.0-50.0)	48.8 (45.0-52.5)	52.3 (48.1-56.4)	51.4 (47.5-55.2)	53.3 (48.7-57.9)	51.5 (48.4-54.6)	53.7 (51.2-56.3)	<.001	7.6 (3.1 to 12.2)
Non-Hispanic Black	58.7 (52.4-64.9)	55.6 (50.1-61.1)	57.8 (51.2-64.4)	57.2 (51.4-62.9)	56.4 (51.3-61.5)	56.1 (51.9-60.2)	60.9 (57.3-64.6)	59.1 (54.9-63.2)	60.2 (55.9-64.5)	61.3 (56.7-65.9)	.10	2.6 (-5.2 to 10.5)
Mexican American	57.6 (50.8-64.3)	58.1 (50.0-66.2)	56.0 (50.7-61.2)	60.4 (56.9-64.0)	62.8 (57.5-68.0)	64.5 (60.3-68.6)	63.3 (56.4-70.3)	68.4 (64.7-72.0)	67.2 (62.4-71.9)	67.4 (62.5-72.3)	<.001	9.8 (1.4 to 18.2)
P value for interaction						.17						
FDR *P* value for interaction						.41						
**Education level**												
Less than high school	44.5 (39.9-49.0)	40.8 (35.7-45.8)	40.8 (34.6-47.0)	44.0 (39.5-48.5)	42.6 (36.5-48.7)	43.0 (36.7-49.2)	41.6 (36.0-47.1)	43.9 (39.8-48.1)	49.8 (42.2-57.5)	46.2 (41.2-51.1)	.12	1.7 (-5.0 to 8.4)
High school or equivalent	42.9 (38.6-47.2)	42.3 (37.7-46.9)	42.6 (39.3-45.9)	45.9 (42.6-49.3)	48.6 (43.7-53.5)	47.4 (42.9-52.0)	47.2 (41.6-52.8)	49.1 (43.5-54.6)	43.8 (40.4-47.3)	50.1 (47.2-53.0)	<.001	7.2 (1.9 to 12.5)

© 2023 Li Y et al. *JAMA Network Open*.

	Estimated prevalence of never smoking (95% CI)[a]											Difference, 2017-March 2020 vs 1999-2000 (95% CI)[b]
	**1999-2000**	**2001-2002**	**2003-2004**	**2005-2006**	**2007-2008**	**2009-2010**	**2011-2012**	**2013-2014**	**2015-2016**	**2017-March 2020**	***P* value for trend**	
College or above	55.4 (52.0-58.8)	58.0 (52.7-63.4)	53.3 (51.0-55.6)	54.4 (51.5-57.4)	56.7 (53.2-60.2)	61.6 (57.3-65.8)	60.2 (55.9-64.5)	62.3 (58.5-66.0)	60.7 (58.4-63.1)	62.9 (61.0-64.8)	<.001	7.5 (3.5 to 11.4)
P value for interaction						.26						
FDR *P* value for interaction						.48						
**Family income–poverty ratio level[c]**												
<1.30	45.8 (40.3-51.2)	46.7 (39.3-54.2)	43.1 (38.7-47.5)	44.1 (40.4-47.8)	44.3 (37.6-50.9)	47.0 (41.3-52.7)	50.0 (43.7-56.3)	47.3 (42.5-52.1)	49.1 (45.0-53.3)	48.6 (44.7-52.5)	.08	2.8 (-3.9 to 9.6)
1.30-3.49	49.3 (43.8-54.8)	45.5 (39.3-51.6)	46.7 (44.2-49.1)	48.3 (45.6-50.9)	52.1 (46.4-57.9)	53.3 (49.9-56.6)	52.2 (48.7-55.6)	54.9 (48.6-61.3)	53.4 (49.5-57.3)	54.7 (50.4-59.1)	<.001	5.5 (-1.6 to 12.5)
≥3.50	52.3 (48.9-55.6)	56.4 (51.3-61.6)	52.6 (49.6-55.6)	55.0 (51.5-58.6)	55.5 (51.6-59.4)	60.5 (55.2-65.7)	59.4 (54.6-64.2)	62.1 (57.4-66.9)	61.1 (57.6-64.6)	63.3 (60.8-65.7)	<.001	11.0 (6.8 to 15.2)
P value for interaction						.10						
FDR *P* value for interaction						.39						

Abbreviations: FDR, false discovery rate.

[a] Estimated prevalence were adjusted for NHANES survey weights.

[b] Values may not equal the difference between the first cycle and last cycle estimates because of rounding.

[c] The variable was calculated by dividing family income by the poverty guidelines. A higher ratio represents a higher level of income.

© 2023 Li Y et al. *JAMA Network Open*.

eTable 9. Crude Trends in Estimated Prevalence of Moderate or Lighter Alcohol Consumption by Age Group, Sex, Race and Ethnicity, Educational Level, and Income, 1999 to March 2020[a]

	Estimated prevalence of moderate or lighter alcohol consumption (95% CI)[b]											
	**1999-2000**	**2001-2002**	**2003-2004**	**2005-2006**	**2007-2008**	**2009-2010**	**2011-2012**	**2013-2014**	**2015-2016**	**2017-March 2020**	***P* value for trend**	**Difference, 2017-March 2020 vs 1999-2000 (95% CI)[c]**
**Age group, y**												
20-34	90.7 (88.7-92.7)	90.8 (87.5-94.1)	91.5 (88.8-94.1)	88.3 (84.6-92.1)	89.9 (87.3-92.5)	89.8 (88.0-91.5)	90.3 (87.4-93.3)	91.5 (89.8-93.2)	91.1 (88.2-94.0)	92.8 (91.0-94.6)	.11	2.1 (-0.6 to 4.8)
35-49	89.4 (86.3-92.4)	91.3 (89.3-93.4)	91.5 (89.0-94.1)	90.4 (89.0-91.8)	90.5 (88.0-92.9)	89.4 (87.3-91.6)	90.1 (87.0-93.2)	89.9 (86.7-93.0)	91.8 (89.2-94.3)	91.3 (88.3-94.3)	.68	1.9 (-2.4 to 6.2)
50-64	90.8 (87.5-94.1)	90.8 (87.5-94.2)	92.0 (90.3-93.7)	89.9 (87.3-92.6)	92.8 (89.9-95.8)	90.4 (88.0-92.9)	84.9 (81.2-88.5)	90.2 (88.6-91.8)	88.9 (86.4-91.5)	90.7 (88.2-93.2)	.23	-0.1 (-4.3 to 4.1)
≥65	94.6 (92.7-96.5)	96.4 (95.1-97.7)	95.4 (93.4-97.3)	96.0 (95.1-96.9)	96.0 (94.7-97.2)	94.8 (92.9-96.8)	94.6 (92.6-96.6)	95.2 (92.9-97.4)	95.4 (92.9-97.8)	95.3 (93.8-96.8)	.69	0.7 (-1.8 to 3.2)
P value for interaction						.23						
FDR *P* value for interaction						.48						
**Sex**												
Women	92.7 (90.2-95.2)	94.4 (92.3-96.5)	94.1 (92.5-95.8)	92.9 (90.8-94.9)	93.1 (90.5-95.8)	92.2 (90.5-94.0)	90.0 (87.0-93.0)	92.0 (90.2-93.7)	92.6 (91.0-94.2)	93.8 (92.6-94.9)	.43	1.1 (-1.7 to 3.8)
Men	89.2 (87.1-	89.3 (87.5-	90.4 (88.9-	88.4 (86.7-	90.4 (88.4-	89.2 (87.3-	89.0 (87.2-	90.8 (89.2-	90.6 (88.2-	90.9 (89.2-	.09	1.7 (-1.0 to 4.5)

© 2023 Li Y et al. *JAMA Network Open*.

	Estimated prevalence of moderate or lighter alcohol consumption (95% CI)[b]										*P* value for trend	Difference, 2017-March 2020 vs 1999-2000 (95% CI)[c]
	1999-2000	2001-2002	2003-2004	2005-2006	2007-2008	2009-2010	2011-2012	2013-2014	2015-2016	2017-March 2020		
	91.4)	91.0)	91.9)	90.0)	92.4)	91.0)	90.7)	92.5)	93.0)	92.6)		
P value for interaction						.09						
FDR *P* value for interaction						.38						
**Race/ethnicity**												
Non-Hispanic White	89.6 (87.3-91.8)	91.0 (89.5-92.6)	92.2 (90.8-93.7)	89.2 (87.2-91.3)	91.8 (89.9-93.8)	89.8 (87.8-91.8)	88.0 (85.8-90.2)	90.3 (88.7-92.0)	90.2 (87.8-92.6)	91.4 (89.9-93.0)	.94	1.8 (-0.9 to 4.6)
Non-Hispanic Black	95.1 (93.5-96.6)	92.9 (90.3-95.6)	93.4 (91.1-95.7)	93.5 (91.2-95.8)	90.9 (88.6-93.2)	91.7 (88.9-94.4)	91.4 (89.2-93.5)	92.2 (90.0-94.4)	93.2 (90.9-95.5)	92.4 (91.2-93.6)	.13	-2.7 (-4.6 to -0.7)
Mexican American	93.7 (91.9-95.5)	93.9 (91.6-96.2)	93.9 (92.0-95.7)	94.6 (92.0-97.2)	89.7 (85.6-93.8)	92.8 (90.9-94.6)	94.5 (92.3-96.6)	94.5 (91.3-97.7)	94.2 (92.4-96.0)	95.2 (93.6-96.9)	.10	1.6 (-0.9 to 4.0)
P value for interaction						.08						
FDR *P* value for interaction						.38						
**Education level**												
Less than high school	91.6 (90.2-93.0)	92.5 (90.3-94.7)	91.7 (89.9-93.5)	89.2 (86.6-91.9)	90.7 (87.2-94.1)	88.7 (85.6-91.7)	93.0 (90.5-95.5)	93.0 (90.1-95.9)	93.5 (92.0-95.0)	91.8 (87.6-96.0)	.47	0.1 (-4.3 to 4.6)
High school or equivalent	90.3 (87.6-93.0)	90.4 (88.2-92.5)	91.4 (88.1-94.6)	91.1 (88.2-94.1)	89.1 (86.2-92.1)	89.2 (86.2-92.2)	86.6 (82.5-90.6)	90.5 (88.0-93.0)	89.5 (84.8-94.1)	93.6 (91.6-95.7)	.20	3.3 (-0.1 to 6.7)

© 2023 Li Y et al. *JAMA Network Open*.

	Estimated prevalence of moderate or lighter alcohol consumption (95% CI)[b]											
	1999-2000	2001-2002	2003-2004	2005-2006	2007-2008	2009-2010	2011-2012	2013-2014	2015-2016	2017-March 2020	*P* value for trend	Difference, 2017-March 2020 vs 1999-2000 (95% CI)[c]
College or above	91.1 (88.9-93.2)	92.4 (90.5-94.2)	92.9 (91.4-94.4)	90.9 (89.1-92.7)	93.5 (91.6-95.4)	91.9 (90.3-93.5)	89.6 (87.4-91.7)	91.4 (90.0-92.7)	91.9 (90.6-93.2)	91.9 (90.7-93.2)	.63	0.9 (-1.6 to 3.4)
P value for interaction						.25						
FDR *P* value for interaction						.48						
**Family income–poverty ratio level[d]**												
<1.30	92.9 (91.4-94.5)	94.2 (92.2-96.2)	91.9 (90.1-93.7)	90.0 (87.1-92.8)	90.6 (88.0-93.3)	88.1 (84.4-91.8)	88.7 (85.4-92.1)	92.5 (89.8-95.2)	93.2 (91.2-95.1)	93.6 (92.3-94.8)	.65	0.6 (-1.4 to 2.7)
1.30-3.49	90.3 (87.5-93.2)	92.2 (90.5-94.0)	93.1 (90.9-95.4)	91.5 (88.8-94.3)	92.7 (91.3-94.0)	91.8 (89.5-94.1)	90.4 (87.9-93.0)	91.8 (89.2-94.3)	92.2 (89.7-94.6)	92.9 (91.0-94.8)	.52	2.6 (-0.9 to 6.0)
≥3.50	90.8 (89.0-92.7)	90.8 (88.6-93.0)	92.2 (90.1-94.3)	90.5 (88.5-92.5)	91.5 (88.6-94.3)	90.5 (88.7-92.4)	89.1 (85.8-92.3)	90.3 (88.8-91.9)	89.4 (86.8-92.0)	91.8 (90.0-93.6)	.78	1.0 (-1.6 to 3.6)
P value for interaction						.73						
FDR *P* value for interaction						.79						

Abbreviations: FDR, false discovery rate.

[a] Moderate or lighter alcohol consumption was defined as ≤14 drinks/week for men or ≤7 drinks/week for women.

[b] Estimated prevalence were adjusted for NHANES survey weights.

[c] Values may not equal the difference between the first cycle and last cycle estimates because of rounding.

[d] The variable was calculated by dividing family income by the poverty guidelines. A higher ratio represents a higher level of income.

© 2023 Li Y et al. *JAMA Network Open*.

eTable 10. Crude Trends in Estimated Prevalence of Healthy Diet by Age Group, Sex, Race and Ethnicity, Educational Level, and Income, 1999 to March 2020[a]

	Estimated prevalence of healthy diet (95% CI)[b]											
	**1999-2000**	**2001-2002**	**2003-2004**	**2005-2006**	**2007-2008**	**2009-2010**	**2011-2012**	**2013-2014**	**2015-2016**	**2017-March 2020**	***P* value for trend**	**Difference, 2017-March 2020 vs 1999-2000 (95% CI)[c]**
**Age group, y**												
20-34	9.3 (6.3-12.3)	12.0 (9.5-14.6)	11.8 (8.8-14.9)	14.2 (10.4-18.0)	16.6 (11.8-21.5)	19.6 (15.2-24.0)	22.8 (18.7-27.0)	20.2 (17.0-23.3)	23.5 (17.7-29.2)	18.7 (14.4-23.0)	<.001	9.4 (4.1 to 14.7)
35-49	18.6 (13.5-23.8)	19.7 (15.9-23.5)	15.9 (13.7-18.2)	19.7 (16.0-23.5)	18.4 (12.6-24.2)	22.5 (19.1-26.0)	23.2 (18.4-28.0)	25.0 (22.3-27.8)	24.0 (19.0-29.0)	22.3 (18.7-26.0)	.004	3.7 (-2.6 to 10.0)
50-64	23.8 (19.0-28.6)	26.8 (23.7-29.9)	25.8 (22.6-29.0)	23.2 (19.4-26.9)	30.0 (24.3-35.8)	32.5 (27.8-37.2)	33.8 (28.9-38.7)	30.6 (25.6-35.7)	27.3 (22.5-32.1)	26.9 (23.2-30.6)	.13	3.0 (-3.1 to 9.1)
≥65	31.4 (26.2-36.5)	33.2 (29.0-37.4)	31.1 (27.9-34.2)	35.4 (29.9-40.9)	33.3 (28.2-38.4)	38.4 (34.6-42.3)	39.0 (31.4-46.5)	36.2 (31.8-40.6)	34.0 (29.5-38.4)	31.6 (27.2-35.9)	.80	0.2 (-6.6 to 7.0)
P value for interaction	<.001											
FDR *P* value for interaction	.004											
**Sex**												
Women	21.1 (16.9-25.3)	23.8 (21.0-26.7)	21.0 (17.9-24.2)	23.5 (20.8-26.3)	26.5 (21.1-31.9)	30.1 (28.1-32.1)	31.7 (27.6-35.7)	29.5 (27.3-31.8)	29.9 (25.1-34.7)	27.3 (23.9-30.7)	<.001	6.3 (0.8 to 11.7)
Men	17.4 (14.4-	18.8 (16.0-	18.5 (16.6-	20.0 (17.2-	19.9 (15.8-	24.1 (21.6-	26.2 (23.1-	25.0 (22.1-	23.7 (20.0-	21.5 (18.5-	<.001	4.1 (-0.1 to 8.4)

© 2023 Li Y et al. *JAMA Network Open*.

	Estimated prevalence of healthy diet (95% CI)[b]											
	1999-2000	2001-2002	2003-2004	2005-2006	2007-2008	2009-2010	2011-2012	2013-2014	2015-2016	2017-March 2020	*P* value for trend	Difference, 2017-March 2020 vs 1999-2000 (95% CI)[c]
	20.4)	21.7)	20.4)	22.9)	24.1)	26.5)	29.3)	28.0)	27.3)	24.5)		
P value for interaction						.44						
FDR *P* value for interaction						.68						
**Race/ethnicity**												
Non-Hispanic White	19.4 (15.3-23.4)	22.5 (19.6-25.3)	20.8 (18.6-23.0)	22.6 (20.1-25.0)	23.8 (17.5-30.0)	29.0 (26.8-31.3)	29.6 (25.4-33.8)	27.6 (24.7-30.4)	27.4 (23.8-30.9)	24.3 (20.0-28.5)	.002	4.9 (-1.0 to 10.9)
Non-Hispanic Black	13.3 (9.4-17.1)	16.1 (11.3-20.8)	13.5 (10.0-17.1)	17.0 (13.0-21.0)	18.3 (15.0-21.7)	18.1 (13.7-22.4)	20.4 (14.4-26.5)	20.2 (16.3-24.0)	18.3 (14.8-21.7)	18.1 (15.7-20.4)	.009	4.8 (0.3 to 9.4)
Mexican American	22.0 (18.6-25.4)	21.7 (18.3-25.1)	16.1 (12.0-20.2)	22.1 (17.2-27.0)	22.2 (18.2-26.3)	24.3 (21.4-27.1)	23.7 (19.1-28.4)	24.8 (19.8-29.8)	19.3 (14.2-24.4)	23.4 (18.2-28.7)	.32	1.4 (-4.9 to 7.7)
P value for interaction						.56						
FDR *P* value for interaction						.76						
**Education level**												
Less than high school	18.1 (15.2-21.1)	14.3 (11.8-16.9)	14.3 (11.3-17.4)	20.7 (16.1-25.2)	16.8 (14.1-19.4)	21.2 (17.2-25.3)	21.7 (18.5-24.9)	18.3 (14.4-22.1)	18.7 (15.0-22.3)	23.2 (18.9-27.5)	.002	5.1 (-0.2 to 10.3)
High school or equivalent	14.6 (11.3-17.8)	16.6 (12.8-20.4)	14.8 (11.9-17.7)	15.3 (11.7-18.8)	17.8 (13.0-22.7)	19.9 (17.3-22.5)	23.7 (18.3-29.1)	18.0 (14.8-21.2)	17.9 (14.6-21.3)	18.0 (14.1-22.0)	.07	3.5 (-1.7 to 8.6)

© 2023 Li Y et al. *JAMA Network Open*.

	Estimated prevalence of healthy diet (95% CI)[b]											
	**1999-2000**	**2001-2002**	**2003-2004**	**2005-2006**	**2007-2008**	**2009-2010**	**2011-2012**	**2013-2014**	**2015-2016**	**2017-March 2020**	***P* value for trend**	**Difference, 2017-March 2020 vs 1999-2000 (95% CI)[c]**
College or above	22.3 (17.7-27.0)	25.8 (22.1-29.4)	23.8 (21.4-26.2)	25.0 (22.9-27.1)	28.4 (23.1-33.7)	31.8 (29.3-34.4)	32.4 (28.6-36.2)	32.6 (30.3-34.9)	31.4 (27.7-35.1)	27.6 (24.4-30.7)	<.001	5.2 (-0.5 to 10.9)
P value for interaction						.64						
FDR *P* value for interaction						.76						
**Family income–poverty ratio level[d]**												
<1.30	17.1 (13.7-20.6)	12.4 (10.7-14.1)	12.5 (8.9-16.2)	17.5 (15.0-20.0)	16.9 (12.5-21.3)	19.0 (16.2-21.9)	20.2 (16.3-24.1)	17.8 (15.1-20.5)	19.1 (16.7-21.6)	18.3 (14.3-22.3)	.009	1.1 (-4.2 to 6.5)
1.30-3.49	15.7 (12.9-18.6)	18.8 (15.0-22.5)	19.5 (15.8-23.1)	20.3 (17.0-23.6)	21.5 (16.1-26.9)	24.3 (22.5-26.2)	25.5 (22.4-28.6)	25.7 (23.1-28.4)	21.8 (18.2-25.5)	22.2 (19.7-24.8)	<.001	6.5 (2.7 to 10.4)
≥3.50	22.7 (17.8-27.5)	27.2 (23.7-30.7)	23.7 (21.9-25.4)	25.0 (21.5-28.4)	27.3 (22.5-32.1)	33.0 (29.6-36.4)	36.8 (31.9-41.6)	34.1 (30.2-37.9)	33.9 (30.5-37.3)	27.8 (23.3-32.3)	<.001	5.1 (-1.5 to 11.8)
P value for interaction						.97						
FDR *P* value for interaction						.97						

Abbreviations: FDR, false discovery rate.

[a] Healthy diet was defined as a Healthy Eating Index-2015 score ≥60.

[b] Estimated prevalence were adjusted for NHANES survey weights.

[c] Values may not equal the difference between the first cycle and last cycle estimates because of rounding.

[d] The variable was calculated by dividing family income by the poverty guidelines. A higher ratio represents a higher level of income.

© 2023 Li Y et al. *JAMA Network Open*.

eTable 11. Crude Trends in Estimated Prevalence of Sufficient Physical Activity by Age Group, Sex, Race and Ethnicity, Educational Level, and Income, 1999 to 2006[a]

	Prevalence of sufficient physical activity (95% CI)[b]					
	**1999-2000**	**2001-2002**	**2003-2004**	**2005-2006**	***P* value for trend**	**Difference, 2005-2006 vs 1999-2000 (95% CI)[c]**
**Age group, y**						
20-34	61.1 (57.5-64.6)	65.6 (59.9-71.3)	61.9 (57.1-66.7)	67.4 (63.7-71.2)	.09	6.4 (1.1 to 11.6)
35-49	55.9 (50.7-61.0)	60.5 (58.2-62.7)	57.0 (53.2-60.7)	65.6 (59.6-71.6)	.045	9.7 (1.7 to 17.8)
50-64	55.0 (48.1-61.9)	57.2 (51.9-62.6)	61.8 (55.9-67.7)	60.3 (54.7-65.8)	.16	5.2 (-3.8 to 14.3)
≥65	47.6 (41.0-54.1)	50.7 (44.2-57.3)	48.7 (44.3-53.1)	52.0 (47.7-56.3)	.37	4.4 (-3.6 to 12.4)
P value for interaction		.74				
FDR *P* value for interaction		.79				
**Sex**						
Women	51.4 (46.6-56.1)	54.9 (50.7-59.1)	54.4 (50.9-57.9)	58.7 (55.3-62.1)	.03	7.3 (1.3 to 13.3)
Men	60.4 (56.5-64.3)	64.5 (61.0-68.0)	62.2 (58.2-66.1)	66.3 (62.6-70.0)	.08	5.9 (0.4 to 11.4)
P value for interaction		.58				
FDR *P* value for interaction		.76				
**Race/ethnicity**						
Non-Hispanic White	60.9 (56.6-65.1)	63.3 (59.8-66.8)	60.7 (57.2-64.3)	64.8 (61.3-68.4)	.30	4.0 (-1.7 to 9.6)
Non-Hispanic Black	40.2 (36.6-43.7)	44.4 (37.8-51.0)	53.7 (48.9-58.5)	51.1 (46.7-55.6)	<.001	11.0 (5.1 to 16.8)
Mexican American	45.4 (42.4-48.3)	46.2 (41.7-50.8)	46.1 (36.5-55.6)	48.5 (41.3-55.8)	.48	3.1 (-4.8 to 11.1)
P value for interaction		.06				
FDR *P* value for interaction		.34				
**Education level**						
Less than high school	37.7 (33.6-41.7)	38.9 (34.3-43.4)	41.8 (36.8-46.7)	43.3 (37.5-49.1)	.09	5.6 (-1.6 to 12.9)
High school or equivalent	52.4 (47.3-57.5)	55.2 (50.7-59.7)	56.5 (52.1-61.0)	57.5 (52.0-63.0)	.17	5.1 (-2.6 to 12.8)
College or above	66.4 (61.0-71.9)	68.0 (65.3-70.7)	63.8 (59.6-68.0)	69.9 (66.8-72.9)	.49	3.4 (-2.9 to 9.8)
P value for interaction		.65				

© 2023 Li Y et al. *JAMA Network Open*.

	Prevalence of sufficient physical activity (95% CI)[b]					
	**1999-2000**	**2001-2002**	**2003-2004**	**2005-2006**	***P* value for trend**	**Difference, 2005-2006 vs 1999-2000 (95% CI)[c]**
FDR *P* value for interaction		.76				
**Family income–poverty ratio level[d]**						
<1.30	41.2 (35.6-46.8)	42.7 (35.8-49.5)	47.1 (42.2-52.0)	50.4 (46.1-54.7)	.01	9.2 (1.9 to 16.4)
1.30-3.49	52.2 (47.4-57.0)	55.2 (52.0-58.4)	56.3 (50.7-61.9)	59.7 (56.3-63.1)	.01	7.5 (1.5 to 13.5)
≥3.50	68.1 (62.7-73.4)	70.5 (66.9-74.1)	66.2 (62.2-70.2)	69.9 (66.1-73.7)	.91	1.8 (-4.9 to 8.5)
P value for interaction		.11				
FDR *P* value for interaction		.39				

Abbreviations: FDR, false discovery rate.

[a] sufficient physical activity was defined as ≥150 minutes/week moderate to vigorous intensity physical activity.

[b] Estimated prevalence was adjusted for NHANES survey weights.

[c] Values may not equal the difference between the first cycle and last cycle estimates because of rounding.

[d] The variable was calculated by dividing family income by the poverty guidelines. A higher ratio represents a higher level of income.

© 2023 Li Y et al. *JAMA Network Open*.

eTable 12. Crude Trends in Estimated Prevalence of Sufficient Physical Activity by Age Group, Sex, Race and Ethnicity, Educational Level, and Income, 2007 to March 2020[a]

	Estimated prevalence of sufficient physical activity (95% CI)[b]							
	**2007-2008**	**2009-2010**	**2011-2012**	**2013-2014**	**2015-2016**	**2017-March 2020**	***P* value for trend**	**Difference, 2017-March 2020 vs 2007-2008 (95% CI)[c]**
**Age group, y**								
20-34	75.2 (71.3-79.2)	73.4 (67.6-79.1)	78.3 (73.7-82.8)	73.8 (70.9-76.6)	81.3 (77.6-85.1)	82.0 (79.0-85.0)	<.001	6.7 (1.7 to 11.7)
35-49	66.2 (62.3-70.2)	66.7 (64.3-69.2)	69.2 (64.0-74.4)	66.7 (63.4-70.0)	70.0 (64.6-75.3)	71.2 (68.6-73.7)	.02	4.9 (0.2 to 9.7)
50-64	61.4 (55.3-67.5)	62.5 (58.9-66.0)	61.3 (57.5-65.2)	58.0 (53.2-62.9)	60.4 (56.4-64.3)	64.8 (61.4-68.2)	.36	3.4 (-3.7 to 10.5)
≥65	47.5 (42.6-52.4)	46.0 (41.8-50.2)	48.7 (45.5-52.0)	47.3 (43.0-51.5)	52.2 (46.8-57.6)	55.5 (51.6-59.4)	<.001	7.9 (1.6 to 14.3)
P value for interaction				.06				
FDR *P* value for interaction				.34				
**Sex**								
Women	57.4 (52.9-61.9)	55.1 (51.3-59.0)	58.2 (54.8-61.6)	56.1 (53.2-59.0)	61.5 (57.1-66.0)	62.3 (59.9-64.6)	.003	4.8 (-0.3 to 10.0)
Men	72.2 (69.1-75.2)	72.8 (69.7-75.9)	73.5 (70.4-76.7)	69.5 (67.1-71.8)	72.3 (69.3-75.3)	76.4 (73.3-79.4)	.08	4.2 (-0.2 to 8.5)
P value for interaction				.45				
FDR *P* value for interaction				.68				
**Race/ethnicity**								
Non-Hispanic White	66.8 (62.2-71.4)	65.9 (62.0-69.7)	67.8 (64.5-71.2)	62.0 (59.6-64.3)	68.6 (64.9-72.3)	70.7 (68.2-73.1)	.06	3.9 (-1.4 to 9.1)
Non-Hispanic Black	54.0	58.8	60.6	62.2	64.3	64.4	.001	10.4

© 2023 Li Y et al. *JAMA Network Open*.

	Estimated prevalence of sufficient physical activity (95% CI)[b]							
	2007-2008	2009-2010	2011-2012	2013-2014	2015-2016	2017-March 2020	*P* value for trend	Difference, 2017-March 2020 vs 2007-2008 (95% CI)[c]
	(47.3-60.7)	(55.7-62.0)	(56.8-64.5)	(58.1-66.3)	(60.5-68.1)	(60.9-67.9)		(2.7 to 18.0)
Mexican American	62.3 (54.6-70.0)	57.9 (50.4-65.4)	63.7 (55.8-71.6)	63.1 (57.3-69.0)	61.1 (55.0-67.3)	72.4 (69.2-75.6)	.002	10.1 (1.6 to 18.5)
P value for interaction				.12				
FDR *P* value for interaction				.39				
**Education level**								
Less than high school	52.5 (48.1-56.9)	52.7 (48.9-56.5)	52.1 (47.0-57.3)	54.2 (50.5-58.0)	50.3 (44.4-56.1)	57.0 (52.1-61.8)	.30	4.5 (-2.1 to 11.1)
High school or equivalent	61.7 (57.9-65.5)	60.7 (57.0-64.5)	64.2 (57.2-71.3)	59.1 (55.6-62.6)	61.3 (56.9-65.7)	67.8 (64.4-71.2)	.01	6.1 (0.9 to 11.2)
College or above	69.9 (66.6-73.2)	68.3 (63.7-73.0)	69.6 (66.4-72.8)	65.7 (63.6-67.9)	71.8 (68.8-74.8)	71.7 (69.4-73.9)	.13	1.8 (-2.3 to 5.8)
P value for interaction				.60				
FDR *P* value for interaction				.76				
**Family income–poverty ratio level[d]**								
<1.30	58.4 (53.8-63.0)	60.7 (56.4-65.0)	62.9 (57.3-68.4)	58.9 (56.3-61.4)	60.9 (56.4-65.4)	63.4 (60.5-66.3)	.16	5.0 (-0.5 to 10.5)
1.30-3.49	61.0 (57.0-65.0)	59.2 (54.1-64.3)	66.0 (61.4-70.6)	60.4 (57.7-63.2)	64.8 (61.0-68.7)	67.6 (64.2-70.9)	.005	6.5 (1.3 to 11.8)
≥3.50	69.9 (66.5-73.4)	70.2 (67.0-73.5)	68.9 (64.9-72.9)	66.5 (62.9-70.2)	73.5 (69.3-77.7)	72.3 (70.0-74.6)	.09	2.3 (-1.9 to 6.6)
P value for interaction				.26				
FDR *P* value for interaction				.48				

Abbreviations: FDR, false discovery rate.

© 2023 Li Y et al. *JAMA Network Open*.

[a] sufficient physical activity was defined as ≥150 minutes/week moderate to vigorous intensity physical activity.

[b] Estimated prevalence was adjusted for NHANES survey weights.

[c] Values may not equal the difference between the first cycle and last cycle estimates because of rounding.

[d] The variable was calculated by dividing family income by the poverty guidelines. A higher ratio represents a higher level of income.

© 2023 Li Y et al. *JAMA Network Open*.

eTable 13. Crude Trends in Estimated Prevalence of Healthy Weight by Age Group, Sex, Race and Ethnicity, Educational Level, and Income, 1999 to March 2020[a]

	Estimated prevalence of healthy weight (95% CI)[b]											
	**1999-2000**	**2001-2002**	**2003-2004**	**2005-2006**	**2007-2008**	**2009-2010**	**2011-2012**	**2013-2014**	**2015-2016**	**2017-March 2020**	***P* value for trend**	**Difference, 2017-March 2020 vs 1999-2000 (95% CI)[c]**
**Age group, y**												
20-34	40.2 (36.0-44.5)	41.1 (37.3-45.0)	39.8 (36.2-43.4)	39.7 (34.8-44.6)	39.3 (35.7-42.8)	39.2 (34.3-44.1)	39.7 (32.5-47.0)	36.5 (32.7-40.3)	35.9 (31.2-40.7)	33.9 (28.7-39.1)	.009	-6.3 (-13.0 to 0.4)
35-49	32.7 (26.6-38.7)	31.9 (29.1-34.8)	31.0 (27.8-34.2)	30.0 (24.2-35.7)	27.4 (23.4-31.4)	27.2 (23.9-30.5)	25.7 (20.6-30.9)	25.4 (21.6-29.2)	22.6 (18.6-26.7)	20.8 (18.0-23.7)	<.001	-11.8 (-18.6 to -5.1)
50-64	27.1 (23.9-30.4)	25.5 (22.6-28.5)	26.6 (22.5-30.7)	23.6 (19.0-28.2)	24.4 (20.9-28.0)	20.3 (16.5-24.1)	24.6 (20.4-28.7)	22.9 (21.0-24.8)	22.6 (18.5-26.7)	20.3 (16.9-23.8)	.003	-6.8 (-11.5 to -2.0)
≥65	29.0 (24.6-33.4)	29.1 (25.2-33.0)	29.0 (25.4-32.6)	28.8 (25.6-32.0)	27.5 (23.9-31.2)	25.3 (22.8-27.7)	29.0 (24.7-33.2)	26.3 (23.6-28.9)	21.5 (17.5-25.5)	22.3 (19.3-25.3)	<.001	-6.7 (-12.0 to -1.3)
P value for interaction						.13						
FDR *P* value for interaction						.39						
**Sex**												
Women	34.5 (30.8-38.2)	35.5 (32.1-38.9)	35.9 (32.6-39.2)	35.6 (32.1-39.1)	32.9 (29.9-35.9)	31.9 (29.8-34.0)	31.4 (27.2-35.7)	31.0 (28.3-33.7)	28.5 (25.2-31.9)	27.6 (25.3-30.0)	<.001	-6.9 (-11.3 to -2.5)
Men	31.5 (28.3-	29.4 (27.4-	28.1 (24.5-	25.8 (22.3-	26.6 (25.0-	24.6 (20.1-	28.3 (25.2-	24.8 (22.5-	23.4 (20.4-	21.5 (18.2-	<.001	-10.0 (-14.7 to -5.4)

© 2023 Li Y et al. *JAMA Network Open.*

	Estimated prevalence of healthy weight (95% CI)[b]											
	**1999-2000**	**2001-2002**	**2003-2004**	**2005-2006**	**2007-2008**	**2009-2010**	**2011-2012**	**2013-2014**	**2015-2016**	**2017-March 2020**	***P* value for trend**	**Difference, 2017-March 2020 vs 1999-2000 (95% CI)[c]**
	34.7)	31.3)	31.8)	29.3)	28.2)	29.0)	31.4)	27.1)	26.4)	24.7)		
P value for interaction						.77						
FDR *P* value for interaction						.79						
**Race/ethnicity**												
Non-Hispanic White	33.9 (29.9-38.0)	33.8 (31.3-36.3)	33.4 (30.9-35.9)	32.3 (28.1-36.5)	30.7 (28.3-33.2)	28.9 (25.6-32.3)	30.8 (26.5-35.1)	28.2 (26.4-30.0)	26.0 (23.4-28.7)	25.5 (22.4-28.5)	<.001	-8.5 (-13.6 to -3.4)
Non-Hispanic Black	29.2 (25.8-32.6)	24.5 (20.5-28.4)	22.7 (19.0-26.5)	22.1 (17.8-26.4)	24.2 (21.5-26.9)	22.3 (18.8-25.7)	23.2 (20.8-25.6)	23.3 (19.5-27.0)	23.6 (19.8-27.5)	21.8 (19.4-24.1)	.03	-7.4 (-11.6 to -3.3)
Mexican American	26.4 (20.4-32.4)	29.9 (24.6-35.3)	24.0 (18.3-29.7)	23.9 (19.2-28.6)	21.8 (17.4-26.1)	19.2 (13.7-24.8)	20.4 (12.7-28.0)	16.6 (12.8-20.4)	17.8 (14.1-21.4)	14.8 (10.9-18.6)	<.001	-11.6 (-18.8 to -4.4)
P value for interaction						.003						
FDR *P* value for interaction						.047						
**Education level**												
Less than high school	25.6 (22.4-28.8)	26.6 (22.2-30.9)	30.2 (24.9-35.6)	30.6 (27.6-33.5)	27.5 (24.6-30.4)	22.1 (18.5-25.8)	27.4 (23.2-31.6)	23.2 (19.9-26.5)	24.7 (19.9-29.4)	22.2 (19.3-25.0)	.01	-3.4 (-7.7 to 0.9)
High school or equivalent	33.7 (28.1-39.4)	30.6 (27.1-34.0)	28.4 (25.3-31.4)	26.7 (22.5-30.8)	28.3 (25.2-31.3)	25.2 (22.0-28.4)	23.6 (18.0-29.2)	26.2 (22.4-29.9)	20.8 (16.6-25.1)	23.3 (20.0-26.6)	<.001	-10.5 (-17.1 to -3.9)

© 2023 Li Y et al. *JAMA Network Open*.

	Estimated prevalence of healthy weight (95% CI)[b]										P value for trend	Difference, 2017-March 2020 vs 1999-2000 (95% CI)[c]
	1999-2000	2001-2002	2003-2004	2005-2006	2007-2008	2009-2010	2011-2012	2013-2014	2015-2016	2017-March 2020		
College or above	36.3 (33.0-39.6)	35.3 (33.2-37.5)	34.5 (31.7-37.3)	32.8 (28.5-37.1)	31.5 (28.9-34.2)	31.2 (27.0-35.4)	32.4 (28.1-36.8)	29.7 (28.0-31.3)	28.0 (25.6-30.4)	25.6 (23.4-27.8)	<.001	-10.7 (-14.7 to -6.7)
P value for interaction						.15						
FDR *P* value for interaction						.40						
**Family income–poverty ratio level[d]**												
<1.30	32.1 (27.1-37.1)	32.0 (28.0-35.9)	36.7 (33.8-39.7)	29.4 (24.7-34.0)	29.6 (26.3-33.0)	26.9 (22.0-31.7)	27.9 (22.1-33.7)	28.8 (25.5-32.0)	24.7 (20.6-28.8)	25.7 (22.8-28.5)	<.001	-6.4 (-12.3 to -0.6)
1.30-3.49	30.3 (25.3-35.3)	30.6 (27.2-34.0)	30.6 (27.5-33.6)	29.1 (24.3-33.9)	28.4 (25.5-31.4)	27.8 (25.4-30.2)	28.2 (24.2-32.2)	24.3 (22.0-26.5)	27.2 (23.0-31.5)	23.2 (20.3-26.2)	<.001	-7.1 (-12.9 to -1.2)
≥3.50	34.6 (30.0-39.1)	35.3 (31.7-38.9)	32.0 (28.4-35.6)	32.7 (28.9-36.5)	31.8 (29.1-34.5)	29.2 (24.2-34.2)	32.8 (27.4-38.2)	30.5 (27.5-33.5)	25.7 (21.9-29.5)	24.6 (21.5-27.8)	<.001	-9.9 (-15.5 to -4.3)
P value for interaction						.68						
FDR *P* value for interaction						.77						

Abbreviations: FDR, false discovery rate.

[a] Healthy weight was defined as body mass index at 18.5-24.9 kg/m^2.

[b] Estimated prevalence were adjusted for NHANES survey weights.

[c] Values may not equal the difference between the first cycle and last cycle estimates because of rounding.

[d] The variable was calculated by dividing family income by the poverty guidelines. A higher ratio represents a higher level of income.

© 2023 Li Y et al. *JAMA Network Open*.
