# Supplementary material for: Trends in Self-Reported Adherence to Healthy Lifestyle Behaviors Among US Adults, 1999 to March 2020
Source: JAMA Netw Open. 2023 Jul 14;6(7):e2323584. doi: 10.1001/jamanetworkopen.2023.23584 (PMC10349344; doi:10.1001/jamanetworkopen.2023.23584)
Supplement: Supplement 2. — Data Sharing Statement [file jamanetwopen-e2323584-s002.pdf]

Data Sharing Statement

Li. Trends in Self-Reported Adherence to Healthy Lifestyle Behaviors Among US Adults, 1999 to March 2020. *JAMA Netw Open*. Published July 14, 2023. doi:10.1001/jamanetworkopen.2023.23584

Data
Data available: Yes
Data types: Deidentified participant data
How to access data: National Health and Nutrition Examination Survey (NHANES) data are available at https://www.cdc.gov/nchs/nhanes/
When available: beginning date: 07-01-2022

Supporting Documents
Document types: None

Additional Information
Who can access the data: anyone requesting the data
Types of analyses: for any purpose
Mechanisms of data availability: without investigator support.
